# Distributional Pattern of Bacteria, Protists, and Diatoms in Ocean according to Water Depth in the Northern South China Sea

**DOI:** 10.1128/spectrum.02759-21

**Published:** 2022-10-12

**Authors:** Shannan Xu, Yong Liu, Zhe Zhang, Youwei Xu, Zhanhui Qi

**Affiliations:** a South China Sea Fisheries Research Institute, Chinese Academy of Fishery Sciences/Key Laboratory of Marine Ranching, Ministry of Agriculture and Rural Affairs/Guangdong Provincial Key Laboratory of Fishery Ecology and Environment, Guangzhou, China; b Southern Marine Science and Engineering Guangdong Laboratory (Guangzhou), Guangzhou, China; c Scientific Observation and Research Field Station of Pearl River Estuary Ecosystem, Guangzhou, China; Huazhong University of Science and Technology

**Keywords:** northern South China Sea, water depth, metagenome-assembled genomes, antibiotic resistance genes, ocean microbiome

## Abstract

Ocean microbiomes provide insightful details about the condition of water and the global impact of marine ecosystems. A fine-scale analysis of ocean microbes may shed light on the dynamics and function of the ocean microbiome community. In this study, we evaluated the changes in the community and function of marine bacteria, protists, and diatoms corresponding to different ocean depths using next-generation sequencing methods. We found that diatoms displayed a potential water-depth pattern in species richness (alpha diversity) and community composition (beta diversity). However, for bacteria and protists, there was no significant relationship between water depth and species richness. This may be related to the biological characteristics of diatoms. The photosynthesis of diatoms and their distribution may be associated with the fluctuating light regime in the underwater climate. Moreover, salinity displayed negative effects on the abundance of some diatom and bacterial groups, which indicates that salinity may be one of the factors restricting ocean microorganism diversity. In addition, compared to the global ocean microbiome composition, function, and antibiotic resistance genes, a water depth pattern due to the fine-scale region was not observed in this study.

**IMPORTANCE** Fine-scale analysis of ocean microbes provides insights into the dynamics and functions of the ocean microbiome community. Here, using amplicon and metagenome sequencing methods, we found that diatoms in the northern South China Sea displayed a potential water-depth pattern in species richness and community composition, which may be related to their biological characteristics. The potential effects of the differences in geographic sites mainly occurred in the diatom and bacterial communities. Moreover, given the correlation between the environmental factors and relative abundance of antibiotic resistance genes (ARGs), the study of ocean ARG distribution patterns should integrate the potential effects of environmental factors.

## INTRODUCTION

The ocean microbiome is a dilute microbiome system that covers most of the earth’s surface. In recent years, several studies have focused on the composition and function of ocean microbes (1–4). Ocean microbes play important roles in decoupled carbon and nitrogen fixation at the global level (1–3). The ocean microbiome community is predictable in terms of its correspondence to seasons, ocean depth, and organic matter (1, 2, 5–7). Research in this field promotes our understanding of the biological functions of ocean microbes at the global level, with studies finding that many factors (biotic and abiotic environmental) impact the ocean microbiome community (2).

Fine-scale analysis may provide insights into the community dynamics and functions of the ocean microbiome. Community variation along geographical gradients is a well-known ecological trend (8, 9). For example, some freshwater bacteria and diatoms display depth-dependent patterns in their communities (10). The total beta diversity and species turnover of bacteria can be explained by spatial, environmental, and biotic variables (10). Thus, two questions arise: what is the pattern of ocean microbes corresponding to the water depth on a fine scale, and what functional changes in ocean microbes are observed in relation to water depth?

Moreover, ocean microbes are potential pools of antibiotic resistance genes (ARGs) (11, 12). The rise in antibiotic resistance in clinical settings is of great concern, and some ARGs in the global ocean confer resistance to some of the most relevant clinical antibiotics (13). To date, several studies have focused on the distribution pattern of ocean ARGs at the global level (13, 14) and in deep oceans (12, 15). Antibiotics have profound effects on the environmental microbiome, and the study of the relationship between ARGs and bacterial communities is likely to provide important data for environmental protection. ARGs display a distance-decay relationship in water bodies in China, and abiotic and biotic factors (e.g., anthropogenic, bacterial, and physicochemical factors) have profound effects on the spatial distribution of ARG composition (16). A previous study found that three physicochemical factors (water depth, temperature, and total phosphorus) were significantly correlated with the normalized abundance of ARGs (16). These indirect effects on ARGs may be caused by physicochemical factors affecting host bacterial abundance and community composition (17). However, the distribution patterns of ARGs in the ocean depths have scarcely been explored on a fine scale. Understanding the biogeographical patterns of ocean ARGs will provide fundamental information for public and natural ecosystem health.

**FIG 1:**
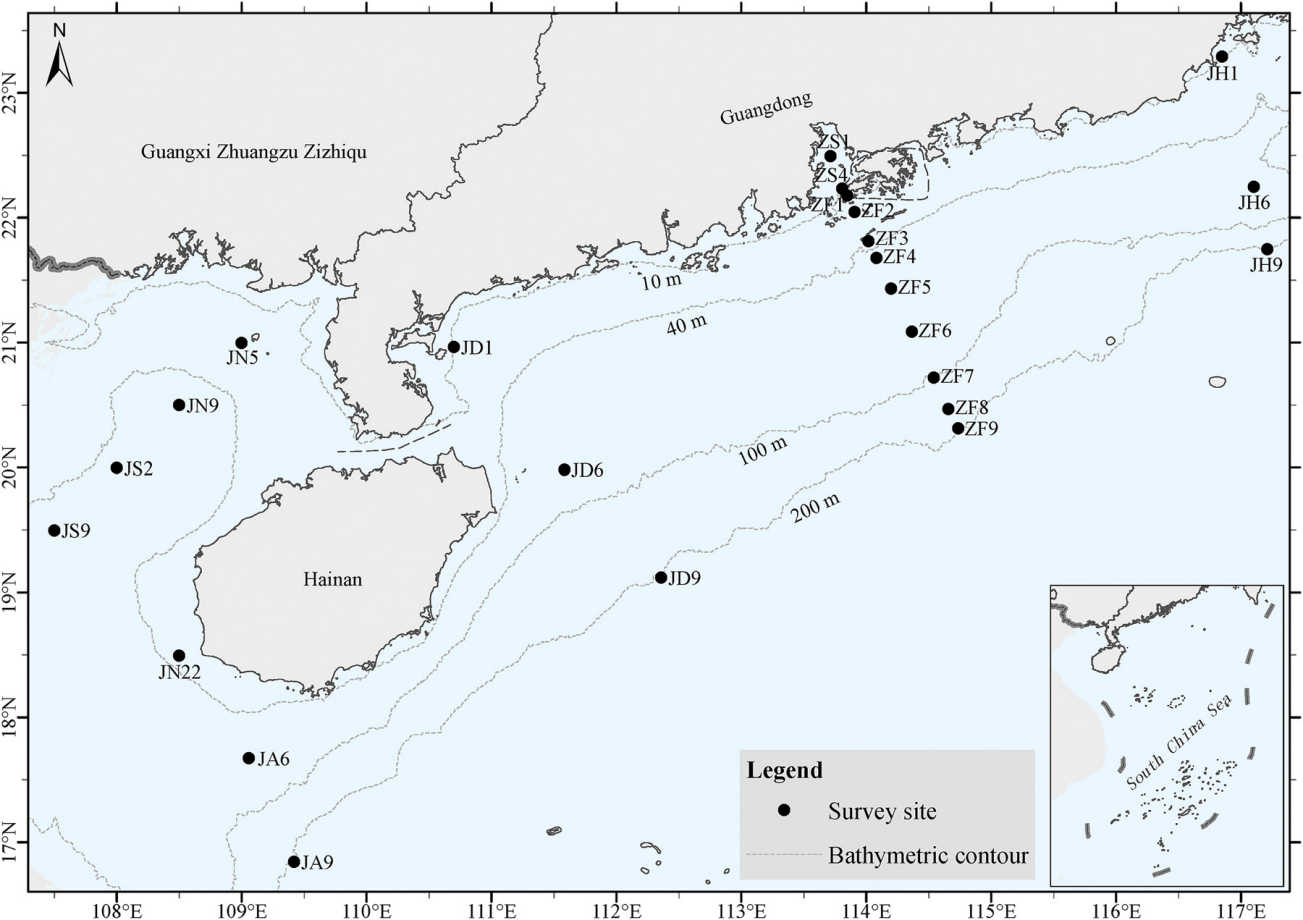
Sample collection sites at water depths of 10, 40, 100, and 200 m.

In this study, we focused on the changes in the community and function of ocean bacteria, protists, and diatoms at different ocean depths (Fig. 1) using next-generation sequencing methods (e.g., amplicon and metagenome sequencing). Moreover, we also measured the physical and chemical properties of water samples obtained during the study. Specifically, we evaluated the alpha and beta diversity of ocean bacteria, protists, and diatoms at different depths. Additionally, we examined different factors to explain water-depth patterns and focused on the functional changes and changes in ARGs in ocean microbes at different depths.

## RESULTS AND DISCUSSION

### Water depth pattern (alpha diversity) and community composition (beta diversity) of diatoms.

**FIG 2:**
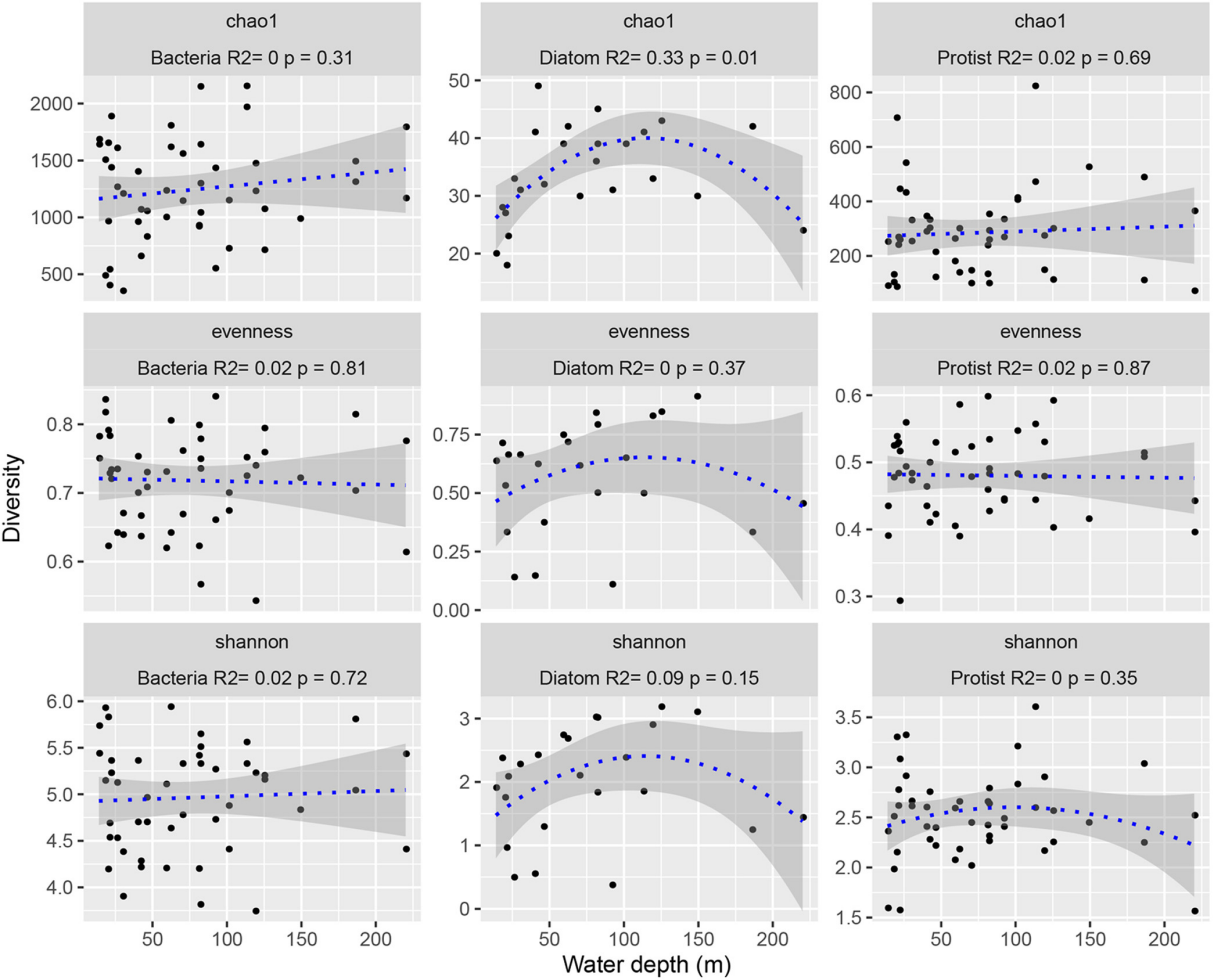
Water-depth patterns for bacteria, diatoms, and protists in terms of alpha diversity. The relationships between water depth and alpha diversity were modeled using linear and quadratic models. The better model was selected based on the lower value of Akaike’s information criteria (53).

**FIG 3:**
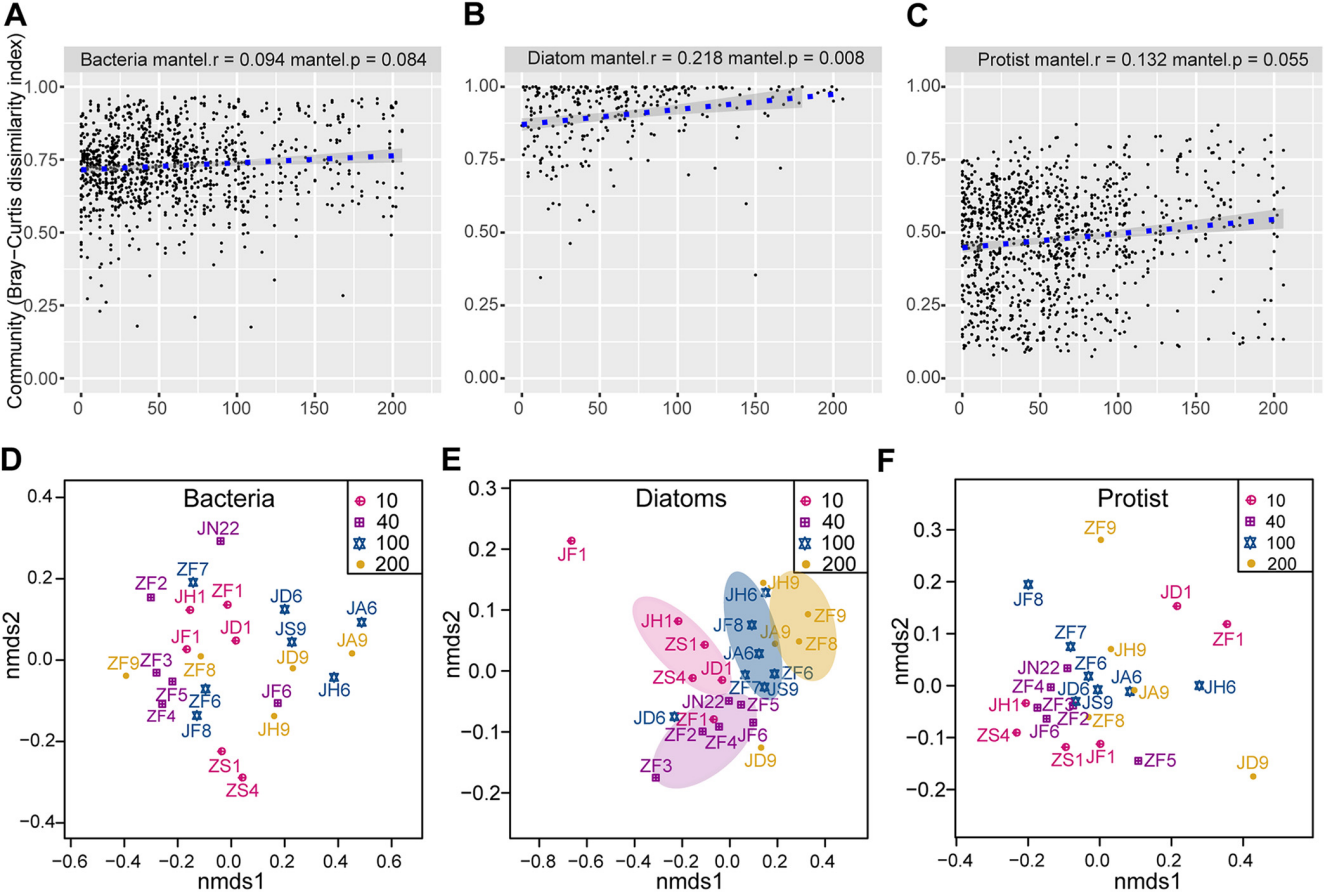
Water-depth patterns for bacteria, diatoms, and protists in terms of beta diversity. The relationships among the community dissimilarities and water depth: (A) bacteria; (B) diatoms; (C) protists. Non-metric multidimensional scaling (NMDS) clustering analysis of the samples from 24 sites based on the Bray-Curtis distance using the community abundance: (D) bacteria; (E) diatoms; and (F) protists.

A significant (*P* = 0.01) relationship was observed between water depth and species richness (Chao1 index) in diatoms, and their species richness exhibited a hump-shaped pattern, with a peak at ~120 m (Fig. 2). However, no significant relationships were observed between water depth and species richness for bacteria or protists, indicative of a random pattern (Fig. 2). Moreover, we found that the Bray-Curtis dissimilarity was positively and significantly related to changes in water depth in relation to diatoms (Mantel test: *r *=* *0.218, *P = *0.008), indicating a distance-decay relationship with water depth and a high turnover rate (Fig. 3B). No significant differences were observed in the distance-decay relationship with water depth and the turnover rates for bacteria (Fig. 3A) (*r *=* *0.094, *P = *0.084) or protists (Fig. 3C) (*r *=* *0.132, *P = *0.055). The non-metric multidimensional scaling (NMDS) results further supported the water-depth pattern for diatoms to some extent (Fig. 3E). RA redundancy analysis (RDA) showed that most of the correlations between the environmental variables and community composition in bacteria, diatoms, and protists were not significant (number of permutations, 999; *P > *0.05) (Table S1), except for the correlation between depth and diatom community (number of permutations, 999; *P = *0.014) (Table S1).

Depth has a profound effect on diatom community composition and diversity in the lake ecosystem (10, 18). In this study, we found that the water depth pattern observed for ocean diatoms did not exist for bacteria and protists at this fine scale. This may be because diatoms are more affected by water conditions and sediment characteristics than bacteria (10, 19–21). In relation to the physical and chemical properties of water, no significant effects on the bacterial and protist communities (beta diversity) were observed. However, principal component analysis (PCA) clustering showed that there was a divergence between the Pearl River Estuary and Beibu Gulf regions based on the Bray-Curtis distance on the environmental factors considered in this study (Fig. S1). We speculated that the differences in the microorganism community and environmental variables between geographic regions may mask the effect of depth when analyzed together. Thus, we re-performed alpha diversity and RDA analyses based on geographic regions (e.g., Pearl River Estuary and Beibu Gulf regions). The results showed no significant relationships between water depth and species richness for all groups (bacteria, protists, and diatoms) in both the Pearl River estuary (Fig. S2) and Beibu Gulf regions (Fig. S3). However, the correlation index (R^2^) for diatoms was larger than that for bacteria and protists, indicating the shallow effects of water depth on diatom alpha diversity (Fig. S2 and S3). These results partially confirmed those obtained by combining all samples (Fig. 2). Furthermore, the RDA results showed that most of the correlations between environmental variables and community composition (bacteria, diatoms, and protists) were not significant (number of permutations, 999; *P > *0.05) (Table S2) for each region. Only one significant correlation was found between salinity and bacteria in the Beibu Gulf region (number of permutations, 999; *P < *0.05) (Table S2). The correlation between salinity and diatoms in the eight Beibu Gulf regions and the Pearl River Estuary was significant (number of permutations, 999; *P < *0.05) (Table S2). The correlation between the other three variables (depth, TP, and SIO_3_) and diatoms was also significant in the Pearl River Estuary region (number of permutations, 999; *P < *0.05) (Table S2). Thus, we deduced that the potential effects of the differences in geographic sites mainly occurred in the diatom and bacterial communities. The alpha and beta diversities of the diatoms showed a partial water-depth pattern. Along with depth, salinity is another important environmental factor influencing diatom and bacterial communities.

**FIG 4:**
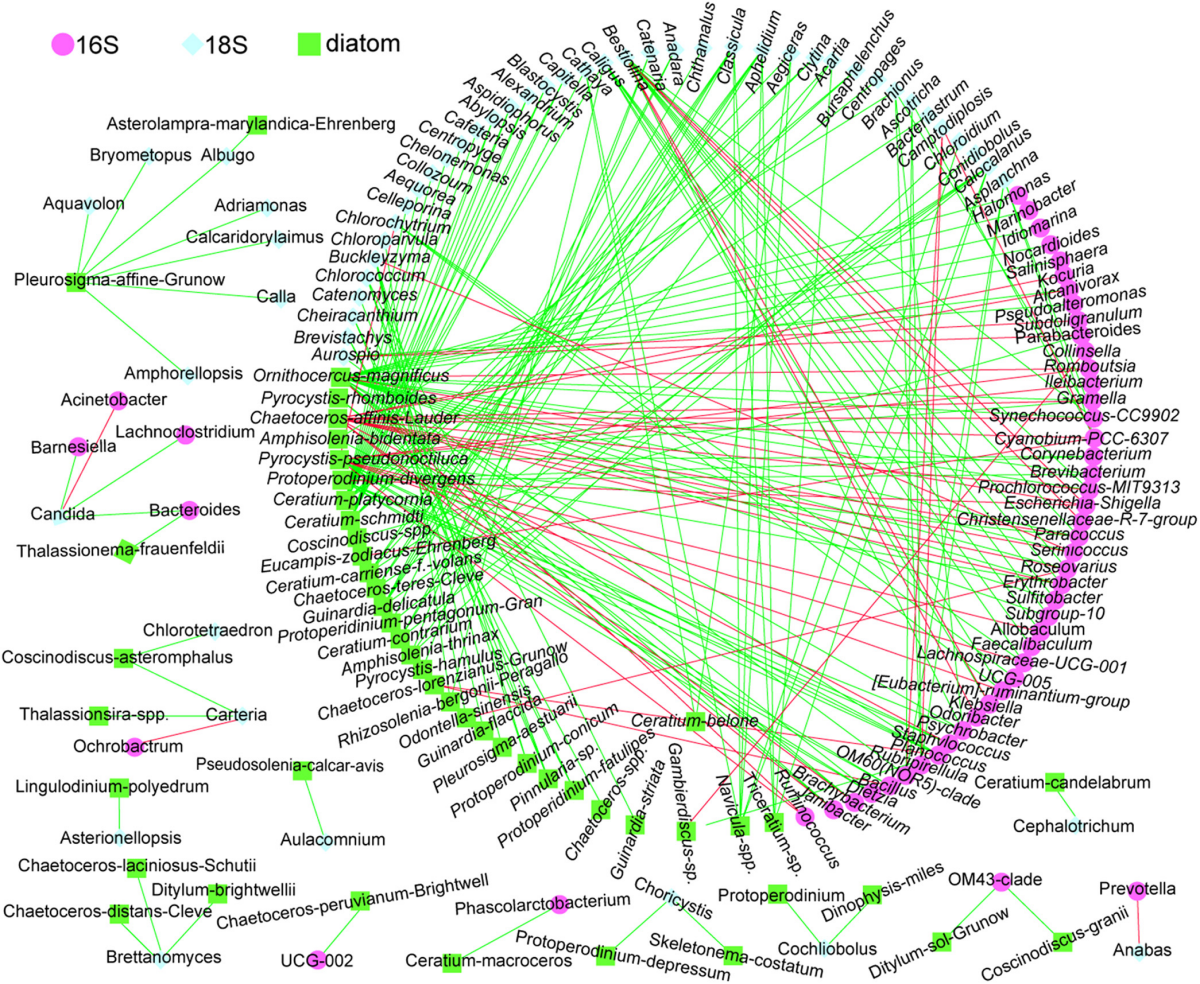
Co-occurrence network among three groups (bacteria, diatoms, and protists). Spearman r = 0.6. Green line: positively significant (0.05); red line: negatively significant (0.05).

In addition, we found that the *r*^2^ (the square of the correlation index) of NH_4−_N was relatively large for all three groups (ocean bacteria, protists, and diatoms), indicating the potential effect of NH_4−_N on the species community. Nitrogen dynamics (e.g., NH_4−_N concentration) affect the abundance of water bacteria and phytoplankton (22, 23). For example, NH_4−_N regeneration has been found to be significantly related to exopolymeric substance (EPS) concentrations, and EPS enhances NH_4−_N regeneration by acting as a carbon source for sea-ice heterotrophs or a substrate for sea-ice bacteria (24). Thus, we speculated that the pattern may not actually be random. This did not correlate with any of the factors examined in this study. Therefore, other environmental factors across different water depths, such as nutrients, pH, and grazing interaction, may further explain the difference between diatoms and bacteria/protists extent (25, 26). Interestingly, the most negative co-occurrence was observed between bacteria and diatoms (Fig. 4). Predation in the aquatic microbial food web is dominated by phagotrophic protists, which maintain the stale grazing-interaction relationship in the ecosystem (27, 28). This may lead to a similar pattern in beta diversity across different water depths. These results indicate that diatoms may be more affected by water depth in the ocean than bacteria or protists.

### Water-depth pattern of diatoms and bacteria.

We further investigated the relationships between groups of bacteria, diatoms, protists, and several environmental variables. We found that three diatom groups (Hemidiscus hardmannianus, Coscinodiscus jonesianus, and Rhizosolenia alata f. *genuina* Gran; Fig. S3 and S4) and one bacterial group (*Prevotella*; Fig. S5 and S6) were positively correlated with water depth. No negative correlations were observed between water depth and the microorganism groups. We found that one diatom group (Ornithocercus magnificus) (Fig. S3) and five bacterial groups (Fig. S5) were negatively correlated with salinity. No positive correlations were observed between salinity and the microorganism groups. No significant correlations were observed between water depth, salinity, and protists (Fig. S7). Furthermore, some groups of bacteria, diatoms, and protists were found to be significantly correlated with ocean nutritive salts (e.g., NO_2−_N, NO_3−_N, NH_4−_N, and PO_4−_P) (Fig. S3, S5, and S7). These results indicate that more diatom groups exhibited a water-depth pattern than bacteria or protist groups. Secondly, some species groups may experience potentially significant effects due to salinity. One previous study found a more diverse bacterial assembly than initially expected at extreme salinities in 14 ephemeral small inland lakes located in the endorheic area of the Monegros Desert (NE Spain) (29). The extremely high dynamism observed in this region may provide a competitive advantage for these versatile (“salt-out”) organisms (29). Overall, the partial water-depth patterns of the diatoms may be related to their biological characteristics. Like algae, diatoms photosynthesize and assimilate inorganic carbon dioxide for conversion into organic substances (30). The spatial distribution of marine diatoms may be associated with the fluctuating light regime in underwater climates (31). This characteristic may explain the high turnover rate associated with increasing water depth.

### Shallow divergence in the function of bacteria with water depth.

**FIG 5:**
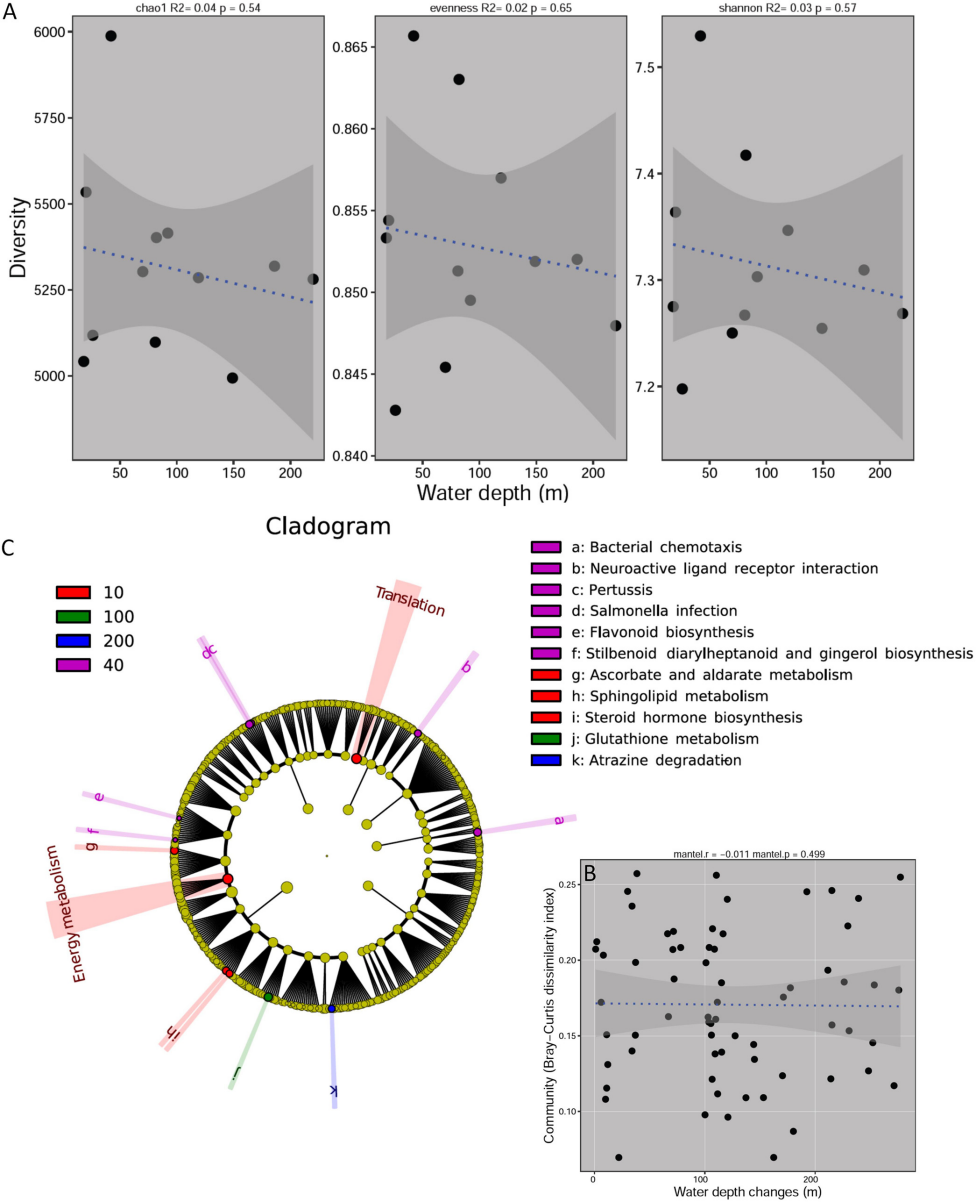
Function of the ocean microbes in this study. (A) Water-depth patterns of bacterial function (putative enzyme composition) in terms of alpha diversity. (B) Relationships between putative bacterial enzyme dissimilarities and water depth. (C) Lefse analysis showing the significant difference in the relative abundance of some KEGG pathways for different water depth gradients: water depths of 10, 40, 100, and 200 m.

Considering the dominance of bacteria in the ocean biomass (2), we chose to focus on the potential differences in their functions across different water depths. We found that functional diversity—alpha diversity (putative enzyme level) and beta diversity (putative enzyme composition)—did not display a water depth pattern (Fig. 5A and B). However, LEfSe analysis of the 24 metagenomes resulted in the identification of a significant difference in the relative abundance of 11 KEGG pathways at four different water depths (Fig. 5C); 6 of them (including flavonoid, stilbenoid, diarylheptanoid, and gingerol biosynthesis) were enriched at a depth of 40 m, while three were enriched at a depth of 10 m. These findings reflect the shallow divergences in ocean bacteria function at different water depths (0 to 200 m) in the coastal area. However, the greatest difference was observed at a depth of 40 m.

**FIG 6:**
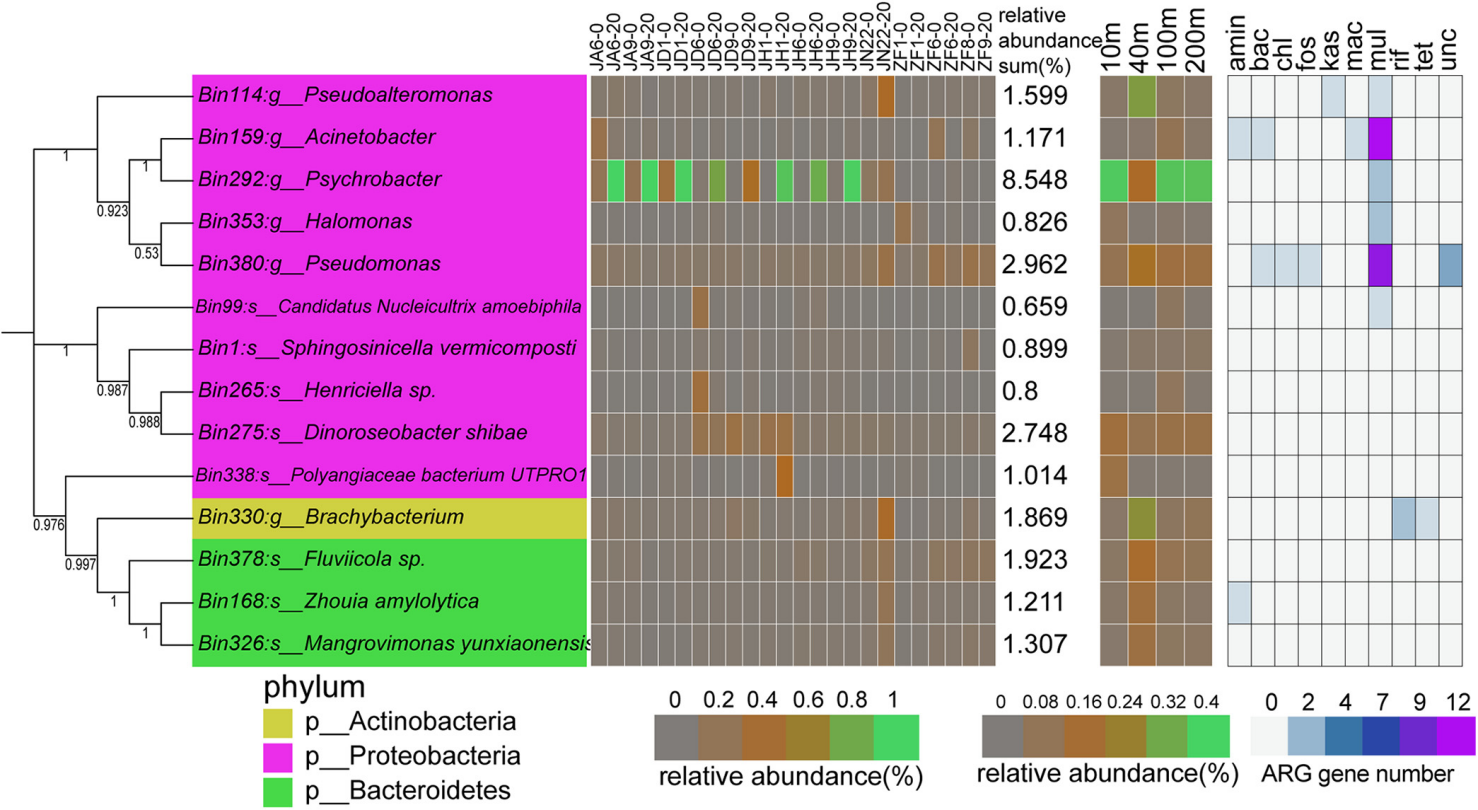
Putative bacterial metagenome-assembled genomes (MAG) by binning among 24 metagenomes: water depths of 10, 40, 100, and 200 m. The heatmap on the left represents the relative abundance of each MAG in each metagenome. The heatmap in the central panel represents the relative abundance of each MAG in each water depth metagenomes. The heatmap on the right represents the number of ARG types in each MAG.

MAG (metagenome assembled genome) level analysis further confirmed the finding that 6 of the 14 putative MAGs (especially Bin330, *Brachybacterium* unclassified and Bin114, *Pseudoalteromonas* unclassified) were enriched in the group at ~40 m depth (Fig. 6). Bin292 (*Psychrobacter* unclassified) was enriched in at all different water depths, except at 40 m. KEGG function analysis showed that many genes were enriched in amino acid, carbohydrate, and energy metabolism (Table S3). MAG Bin330 (*Brachybacterium* unclassified) had the highest number of genes coding for putative enzymes involved in carbohydrate metabolism (Table S2) at KEGG level 2. At KEGG level 3, MAG Bin330 (*Brachybacterium* unclassified) was found to have the highest number of genes encoding putative enzymes involved in amino sugar and nucleotide sugar metabolism. The difference in function between metagenomes and MAGs exists because the metagenomes included all putative functions from the bacterial community in each sample. Thus, we revealed a shallow divergence in bacterial function with increasing water depth.

### High similarity in the composition of ARGs with water depth.

**FIG 7:**
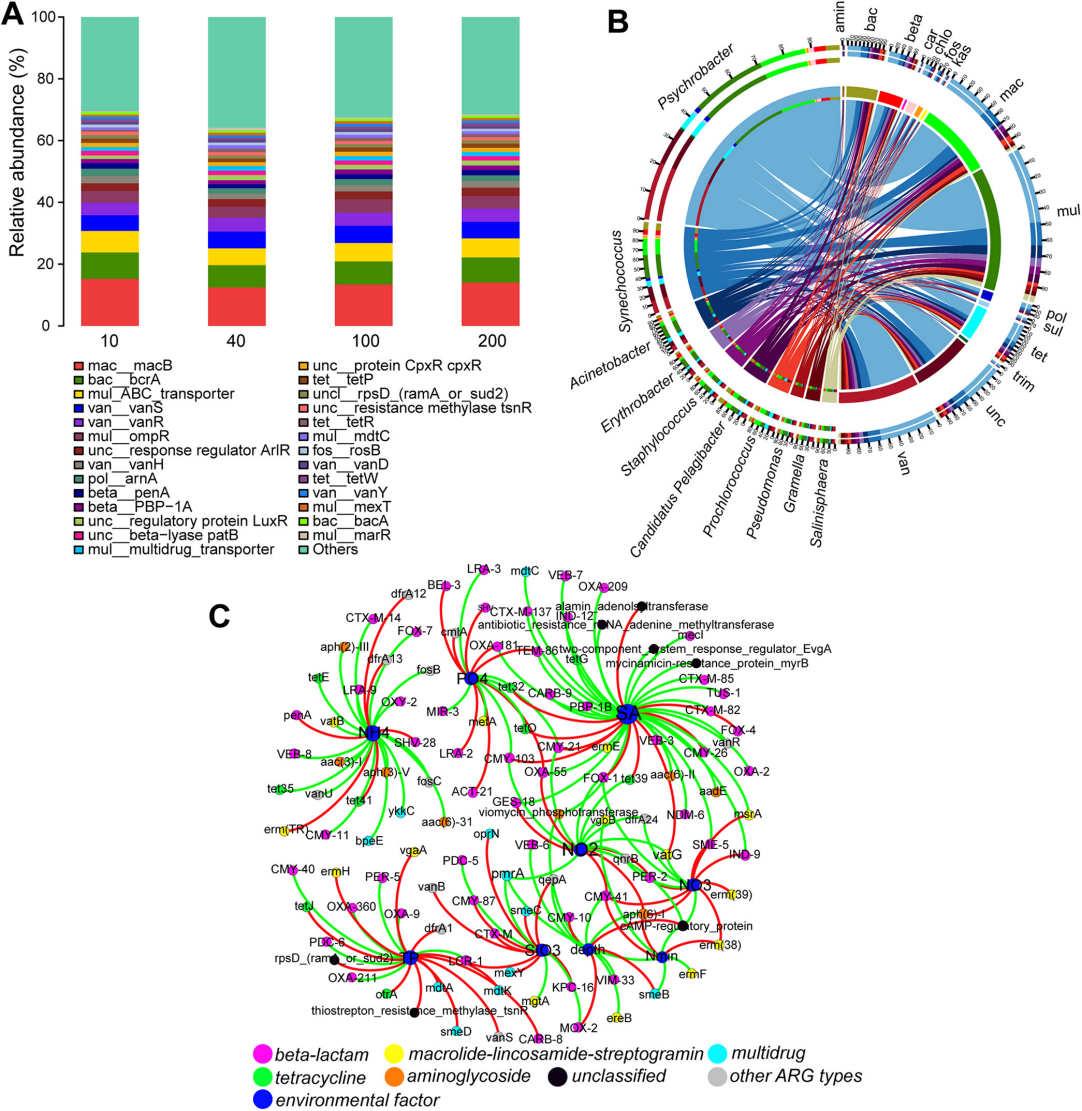
ARGs distribution with water depth. (A) The main ARG types among 24 metagenomes: water depths of 10, 40, 100, and 200 m. (B) The distribution of the ARG type and putative bacterial source using metagenomes. (C) The co-occurrence network displayed the relationship between the relative abundance of ARG types and environmental factors.

**FIG 8:**
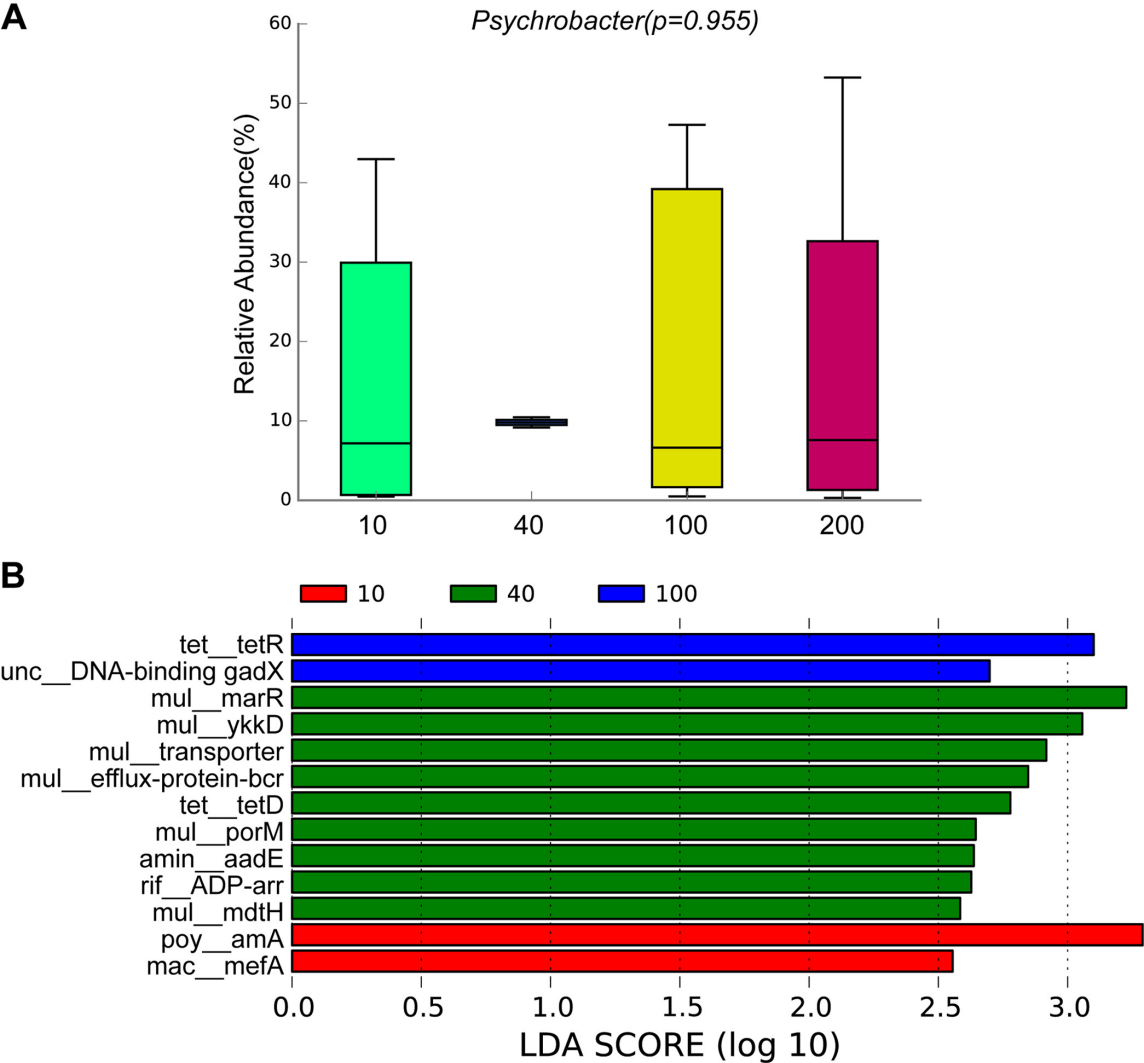
ARGs and their related bacteria. (A) The relative abundance of *Psychrobacter* among different water-depth groups using metagenome data. The pairwise test was based on Kruskal-Wallis test. (B) The LEfSe analysis (*P* ≤ 0.05 and Liner discriminant analysis score ≥ 2.5) displayed the significantly enriched ARG subtypes for each water-depth group.

Here, we found a high similarity in the composition of ARGs (using metagenomes) with water depth (Fig. 7A). For example, the predominant ARG types were macrolide-lincosamide-streptogramin_macB, bacitracin_bcrA, multidrug_ABC transporter, beta-lactam, and vancomycin_vanS. These ARG types mainly originated from *Psychrobacter*, *Synechococcus*, Acinetobacter, *Erythrobacter*, Staphylococcus, and Pseudomonas (Fig. 7B). Multidrug, macrolide-lincosamide-streptogramin, and beta-lactam mainly originated from *Psychrobacter*, *Synechococcus*, Acinetobacter, *Erythrobacter*, Staphylococcus, and Pseudomonas (Fig. 7B). This finding was partially confirmed by ARG analysis of each MAG (Fig. 6). For example, multidrug was the dominant ARG type in these Proteobacteria MAGs, especially the Acinetobacter, Pseudomonas, and Psychrobacter MAGs. Acinetobacter and Pseudomonas MAGs were enriched in different ARG types (Fig. 6). The relative abundance of *Psychrobacter* MAG was high in these metagenomes (Fig. 6), and the relative abundance of *Psychrobacter* was also high in these samples (Fig. 8A). Interestingly, *Psychrobacter* displayed a significant mutual correlation with diatoms and protists (Fig. 4), indicating its important role in the stability of the ocean microbial ecosystem. Proteobacteria is the main bacterial phylum in the ocean (32, 33). *Psychrobacter* is a genus of osmotolerant, psychrophilic, and aerobic bacteria that appear in a wide range of moist, cold, saline habitats, although they also occur in warm and slightly saline habitats (e.g., marine environments) (34–37).

A potential correlation was observed between environmental factors (e.g., salinity and NH_4−_N) and the relative abundance of ARGs (Fig. 7C). Salinity was found to be significantly positively correlated with the relative abundance of bacterial beta-lactam, multidrug, and macrolide-lincosamide-streptogramin subtypes, such as beta-lactam_OXA-55, beta-lactam_OXA-209, beta-lactam_OXA-2, beta-lactam_CTX-M-82, beta-lactam_CTX-M-85, beta-lactam_CTX-M-137, beta-lactam_CMY-21, beta-lactam_CTX-26, multidrug_mdtC, and macrolide-lincosamide-streptogramin_msrA, and a similar pattern existed for NH_4−_N (Fig. 7C). Furthermore, *Synechococcus*, belonging to the phylum Cyanobacteria, is found throughout the world’s oceans (38–41). Thus, ocean nutritive salts may influence the bacterial community (e.g., Proteobacteria and Cyanobacteria). The distribution of ARGs (e.g., multidrug) in the ocean waters evaluated in this study was associated with the abundance of Proteobacteria and Cyanobacteria. However, the transportation of ARGs via aquatic environments is significant, and anthropogenic ARG pollution (e.g., β-lactams, tetracyclines, macrolide-lincosamide-streptogramin, and multidrug) is known to exist in ocean ecosystems (14, 42, 43). Although direct anthropogenic ARG pollution data were not evaluated in this study, we deduced that these abiotic and biotic factors (e.g., ocean nutritive salts, anthropogenic ARG pollution, and the dynamics of the microorganism ecosystem) may contribute to ARG distribution in the ocean.

Moreover, we found significant differences in the relative abundance of some ARG subtypes among different water depths, especially at a depth of 40 m (Fig. 8B). Some multidrug subtypes (e.g., *marR*, transporter, *bcr*, *ykkD*, *oprM*, *mdtH*, and *cmeB*) were found to be significantly enriched in metagenomes at a depth of 40 m. The relative abundance of most MAGs was higher at 40 m than at the other three depths (Fig. 6). Given the dissimilarity in the microorganism community between 40-m depth group and the other three water-depth groups (Fig. 3D to F), we deduced that different bacterial communities may partially lead to the different distribution patterns of some multidrug subtypes. Overall, the ARG composition was highly similar among the different water-depth groups. Previous studies have focused on the distribution pattern of ocean ARGs at the global level (13, 14) and in deep oceans (12, 15). The shallow divergence pattern identified in this study may have been caused by the fine-scale region and similar environmental conditions.

### Conclusion.

We conducted a fine-scale analysis of ocean microbes to gain insights into the dynamics and functions of the ocean microbiome community. We found that diatoms displayed a potential water-depth pattern in species richness (alpha diversity) and community composition (beta diversity), which may be related to their biological characteristics. The photosynthesis of diatoms and their distribution may be associated with the fluctuating light levels in the underwater climate, and salinity displays a negative effect on the abundance of some diatom and bacterial groups. This indicates that salinity may be one of the restricted factors influencing ocean microorganisms. The potential effects of differences in geographic sites were mainly found to occur in the diatom and bacterial communities. Moreover, given the correlation between environmental factors and the relative abundance of ARGs, the study of distribution patterns of ARGs in oceans should integrate the potential effects of environmental factors.

## MATERIALS AND METHODS

### Study area.

This study was conducted on the North Fishing 60011 cruise, a commercial fishing vessel FV Beiyu 60011 (36.8-m length, 6.8-m width, and 3.8-m depth), from 21 September 2018 to 4 October 2018. A total of 24 sampling stations were investigated offshore in the northern South China Sea (Fig. 1 and Table S4). Among the 24 stations, 7 (JN5, JN9, JS2, JS9, JN22, JA6, and JA9) were located in the Beibu Gulf, 11 (ZS1, ZS4, ZF1, ZF2, ZF3, ZF4, ZF5, ZF6, ZF7, ZF8, and ZF9) were located in the Pearl River estuary and adjacent waters, 3 (JD1, JD6, and JD9) were located in the west Guangdong waters, and 3 (JH1, JH6, and JH9) were located in the eastern Guangdong waters (Fig. 1). The water depth in these regions ranged from 10 to 200 m (Fig. 1).

### Sample collection.

Surface water samples at depths of ~0.5 and ~20 m were collected using a pump at each sample site (Table S4). Six water samples were collected at each sample site: three replicates for surface water and three replicates for 20-m (depth) water. Each water sample (approximately 1.5 L) was filtered through a nitrocellulose filter membrane (0.4 μm) (Millipore, Burlington, MA) using a vacuum peristaltic pump (Auto-Science, Tianjing, China). The membranes were stored at −80°C prior to the experiments.

### DNA extraction, PCR amplification, and sequencing.

Microbial DNA was extracted from the filter membranes using the E.Z.N.A. Soil DNA kit (Omega Bio-tek, Norcross, GA) according to the manufacturer’s protocol. The V4 to V5 region of the bacterial 16S rRNA gene (primers: 515F, 5′-GTGCCAGCMGCCGCGG-3′ and 907R, 5′-CCGTCAATTCMTTTRAGTTT-3′) and the V4 region of the protist 18S rRNA gene (TAReF: 5′-CCAGCASCYGCGGTAATTCC-3′ and TAReR: 5′-ACTTTCGTTCTTGATYRA-3′) were amplified by PCR (95°C for 2 min, followed by 25 cycles at 95°C for 30 s, 55°C for 30 s, 72°C for 30 s, and 72°C for 5 min), where the barcode was an eight-base sequence unique to each sample (Table S5). The protocol for the MiSeq platforms was performed as previously described (44). PCR was performed in triplicate in a 20-μL mixture containing 4 μL of 5× FastPfu Buffer, 2 μL of 2.5 mM dNTPs, 0.8 μL of each primer (5 μM), 0.4 μL of FastPfu Polymerase, and 10 ng of template DNA. Amplicons were extracted from 2% agarose gels, purified using the AxyPrep DNA Gel Extraction Kit (Axygen Biosciences, Union City, CA), and quantified using QuantiFluor -ST (Promega, Madison, WI).

The purified PCR products were quantified using Qubit 3.0, and 24 amplicons with different barcodes were mixed in equal proportion. The pooled PCR product was used to construct an Illumina Pair-End library (TruSeq DNA PCR-free sample preparation kit; Illumina, San Diego, CA), according to the instructions for Illumina genomic DNA library preparation. The amplicon library was paired-end sequenced (2 × 250 bp) on an Illumina Hiseq2500 platform (Mingke Biotechnology [Hangzhou] Co. Ltd., Zhejiang, China) according to standard protocols (44).

### Processing of sequencing data.

Raw fastq files were first trimmed using in-house Perl scripts according to the barcode sequence information (for each sample) using the following criteria: (i) the 250-bp (base pair) reads were truncated at any site with an average quality score of <20 over a sliding window of 10 bp; (ii) truncated reads shorter than 50 bp were discarded; (iii) the reads for each sample were extracted based on the barcode sequences (Table S3) and reads containing ambiguous characters were removed; and (iv) pair-end reads which overlapped by over 10 bp were merged. Reads that could not be merged were excluded.

Operational taxonomic units (OTUs) were clustered with a cutoff of 97% similarity using UPARSE (version 7.1; http://drive5.com/uparse/) (45), and chimeric sequences were identified and removed using UCHIME (46). The phylogenetic affiliation of each 16S rRNA gene sequence was analyzed using the RDP Classifier (http://rdp.cme.msu.edu/) against the Silva (SSU132) 16S rRNA database using a confidence threshold of 70% (47). The phylogenetic affiliation of each 18S rRNA gene sequence was analyzed using the PR2 database (http://pr2-database.org/eukref/about/) (48).

### Taxonomic identification of phytoplankton.

A 1-L water sample was preserved with 1% acetic Lugol’s iodine solution as the phytoplankton sample. The samples were processed through a series of settling (allowing them to settle for 48 h) and siphoning steps to obtain a 30-mL concentrate. Species identification and cell counting were performed at 200× or 400× magnification using a 1-mL Sedgewick-Rafter plankton-counting chamber under an inverted microscope (CKX41; Olympus, Tokyo, Japan). At least 300 cells were counted for each sample. Identification was principally performed to the species level when possible. The relevant cell morphologies for phytoplankton species identification usually include shape, flagellums, spines, horns, keels, setae, etc. The numbers and shapes of chromatophore in the cell were also important identification characteristics. Individuals that could not be accurately identified to the species level were classified into the corresponding genus. Only specimens with a diameter of >5 μm were included in the phytoplankton species composition analysis. The group of dinoflagellates with sizes of 5 to 10 μm, primarily including small *Gymnodinium* and cysts, was recorded as nano-dinoflagellates. Phytoplankton identification was performed as described by Guo and Qian (49) and Tomas (50).

### Measurement of physical and chemical properties of water samples.

Temperature and salinity were measured *in situ* using the General Oceanic Sea Bird conductivity, temperature, and depth (CTD). The water was filtered using a GF/F filter (0.7 μm) and frozen at −20°C for subsequent nutrient analysis. Nutrients (e.g., nitrate, nitrite, ammonia, dissolved inorganic phosphate, and reactive silicate) were analyzed using a flow injection analyzer (QuichChem 8500; Lachat Inc., Loveland, CO) according to standard colorimetric techniques (51). Dissolved inorganic nitrogen (DIN) was calculated by obtaining the sum of the nitrate, nitrite, and ammonia compositions in the sample, and the size-fractioned Chl-a concentration was examined at the stations.

### 16S (bacteria), 18S (protists), and diatom analysis.

The mean abundance of bacteria, protists, and diatoms was calculated at the 24 sampling sites for downstream analysis. Rarefaction in Mothur v1.21.1 was used to ensure an even sequencing depth (52). Then, the diversity indices were calculated, including the Chao1, evenness, and Shannon diversity indices. The relationship between water depth and alpha diversity was modeled using linear and quadratic models. The best model was selected based on the lower value of Akaike’s information criterion (53). Beta diversity analysis was performed using an unweighted-UniFrac distance (54) by the vegan 2.0 package in R. The unweighted UniFrac distance for species abundance was used to generate a non-metric multidimensional scaling.

Mantel tests were conducted to examine the Spearman’s rank correlation between water depth and community similarity (bacteria, protists, and diatoms) using Bray-Curtis distance matrices (55) with 999 permutations using the vegan package in R. A multivariate analysis of variance (MANOVA) was conducted to confirm the observed differences.

Spearman’s correlation coefficients were assessed to determine the relationship between microbiota and environmental variables. The correlation was considered significant when the absolute value of Spearman’s rank correlation coefficient (Spearman’s *r*) was >0.6 and statistically significant (*P* < 0.05). All statistical analyses were performed in R. The pheatmap package in R and Cytoscape (http://www.cytoscape.org) were used to visualize relationships through correlation heatmaps and network diagrams, respectively. RA redundancy analysis (RDA) was employed to explore the relationship between environmental factors and microbiota communities (number of permutations, 999).

### Metagenomics analysis.

A total of 24 water samples were collected from 12 sites at different water depths, with two samples collected per site at surface (0 m) and 20 m depth for metagenomic sequencing (Table S4). The raw reads were filtered using Trimmomatic to remove reads less than 50 bp in length, reads with degenerate bases (N’s), and all duplicates defined as sequences; that is, those sequences whose initial 20 nucleotides were identical and showed an overall identity of >97% throughout the length of the shortest read. MEGAHIT (56) was used to assemble clean reads (parameters for minimum contig length [bp]: -min-contig-len 500). MetaProdigal has also been used for gene prediction (57). CD-HIT (58) was used to construct non-redundant gene sets with <90% overlap and <95% shared sequence identity from these gene files. Based on these gene profiles, we used Salmon (59) to map the clean reads to the clean non-redundant gene profile and determine the transcripts per million (TPM) abundance of these non-redundant gene profiles in each metagenome. Finally, clean non-redundant gene sequences were searched against the Kyoto Encyclopedia of Genes and Genomes (KEGG) database using DIAMOND (60). The KEGG Orthology (KO), enzyme commission (EC), and KEGG pathways associated with each sequence were determined. Next, we calculated the relative abundance of KEGG pathways using TPM (61). In addition, we performed BLAST analysis for these genes against the nr database of the National Center for Biotechnology Information (NCBI) using DIAMOND and obtained the putative taxon assignments of these genes per metagenome (60). The annotation of ARGs was performed again using the ARGs-OAP 2.0 database with the default parameters (62). The co-occurrence network between the ARGs and environmental factors was visualized in Cytoscape (63). Linear discriminant analysis effect size (LEfSe) was used to calculate the significant differences in the abundance of ARG subtypes among the three groups (64).

### Metagenome-assembled genome-level analysis.

Clean reads obtained from the 24 metagenomes in this study were combined for binning analysis (BA). Burrows-Wheeler Aligner (BWA) (65) and Samtools (66) were used to map the clean reads to the contigs. We used MetaBAT2 to obtain the contig for each bin (67). Then, CheckM was used for the quality control of each bin, and the resulting high-quality bins (coverage of >80%, contamination rate of <10%) were selected (68). PhyloPhlAn was used to construct a phylogenetic tree of these bins. Salmon (59) was used to map the clean reads to these bins and determine the TPM abundance of these bins in each metagenome. Finally, the bins were searched against the KEGG database using DIAMOND (60). The KO, EC, and KEGG pathways associated with each sequence were determined. Finally, the relative abundance of the KEGG pathways was calculated using TPM (61), and the annotation of the ARGs for each MAG was performed again using the ARGs-OAP 2.0 database with the default parameters (62).

### Data availability.

The raw data sets were deposited in NCBI with the accession number PRJNA680010.
