# Supplementary material for: Distributional Pattern of Bacteria, Protists, and Diatoms in Ocean according to Water Depth in the Northern South China Sea
Source: Microbiol Spectr. 2022 Oct 12;10(6):e02759-21. doi: 10.1128/spectrum.02759-21 (PMC9769685; doi:10.1128/spectrum.02759-21)
Supplement: Supplemental file 1 — Supplemental material. Download spectrum.02759-21-s0001.pdf, PDF file, 1.3 MB [file spectrum.02759-21-s0001.pdf]

Supporting information

Table S1 RDA analysis of the relationship between the environmental factor and community composition (bacteria, diatoms, and protists)

Bacteria

	RDA1	RDA2	r^2	*Pr*(>*r*)
NO_2	-0.00304	1.00000	0.08	0.391
NO_3	-0.0861	0.99629	0.0681	0.447
NH_4	-0.99995	0.00997	0.0178	0.815
Nmin	-0.0885	0.99608	0.0695	0.444
PO_4	0.29137	0.95661	0.0351	0.675
SIO_3	0.06997	0.99755	0.0387	0.633
TP	0.98132	-0.1924	0.1246	0.258
SA	-0.79613	-0.60512	0.1628	0.142
depth	-0.99703	-0.07699	0.1905	0.108

Diatoms

	RDA1	RDA2	r^2	*Pr*(>*r*)
NO_2	-0.48244	0.87593	0.074	0.318
NO_3	-0.54491	0.8385	0.036	0.444
NH_4	0.83326	-0.55288	0.0033	0.968
Nmin	-0.53714	0.84349	0.0382	0.431
PO_4	-0.39966	0.91666	0.1065	0.271
SIO_3	-0.44607	0.895	0.2011	0.09
TP	0.91289	-0.4082	0.1968	0.106
SA	-0.44603	-0.89502	0.0954	0.327
depth	-0.23664	-0.9716	0.3416	0.014*

Protist

	RDA1	RDA2	r^2	*Pr*(>*r*)
NO_2	0.81643	0.57745	0.073	0.326
NO_3	0.68693	0.72672	0.0486	0.442
NH_4	-0.3476	0.93764	0.1929	0.097
Nmin	0.67858	0.73453	0.0524	0.432
PO_4	0.96071	0.27756	0.0531	0.442
SIO_3	0.88207	0.47112	0.0692	0.405
TP	-0.42886	-0.90337	0.0788	0.381
SA	-0.8641	0.50332	0.0544	0.495
depth	-0.9847	0.17425	0.1288	0.244

*, a significant level at 0.05. r^2, the square of the correlation index. *Pr*(>*r*), the *p-value* (number of permutations: 999). SA, salinity. TP, total phosphorus. Nmin, total inorganic nitrogen.

Table S2 RDA analysis of the relationship between the environmental factor and community composition (bacteria, diatoms, and protists) in Pearl River estuary (PR) and Beibu Gulf regions (BG)

Bacteria **PR**

	RDA1	RDA2	r2	Pr(>r)
NO2	-0.95612	0.29296	0.1697	0.426
NO3	-0.90746	0.42014	0.1826	0.429
NH4	0.3193	0.94765	0.0331	0.846
Nmin	-0.89566	0.44473	0.1641	0.474
PO4	-0.21967	-0.97557	0.3987	0.063
SIO3	-0.93344	-0.35873	0.194	0.331
TP	0.74912	-0.66243	0.0688	0.694
SA	-0.06893	0.99762	0.4365	0.048*
depth	-0.52616	0.85039	0.0216	0.901

Bacteria **BG**

	RDA1	RDA2	r2	Pr(>r)
NO2	-0.96128	0.27556	0.106	0.747
NO3	-0.43697	0.89948	0.0279	0.956
NH4	0.10849	-0.9941	0.0076	0.982
Nmin	-0.61241	0.79054	0.0238	0.953
PO4	0.51153	0.85927	0.1946	0.548
SIO3	0.98432	0.17641	0.0431	0.881
TP	-0.97306	0.23055	0.0415	0.901
SA	-0.34998	0.93676	0.0317	0.925
depth	0.35317	0.93556	0.0819	0.784

Diatoms **PR**

	RDA1	RDA2	r2	Pr(>r)
NO2	-0.81031	0.586	0.048	0.748
NO3	-0.98299	0.18366	0.0577	0.711
NH4	0.48022	0.87715	0.0461	0.821
Nmin	-0.94875	0.31604	0.0464	0.747
PO4	0.67619	-0.73673	0.207	0.25
SIO3	-0.60198	-0.79851	0.0539	0.732
TP	0.96833	-0.24969	0.0555	0.749
SA	-0.41618	0.90928	0.5283	0.041*
depth	-0.11938	0.99285	0.074	0.668

Diatoms **BG**

	RDA1	RDA2	r2	Pr(>r)

NO2	-0.82084	0.57116	0.4989	0.132
NO3	-0.72427	0.68952	0.3151	0.324
NH4	0.31949	-0.94759	0.3136	0.341
Nmin	-0.80804	0.58913	0.2174	0.489
PO4	-0.77895	0.62709	0.4331	0.191
SIO3	-0.89541	0.44524	0.6751	0.029*
TP	0.94152	-0.33695	0.7168	0.016*
SA	0.99022	-0.13951	0.7294	0.02*
depth	0.99729	0.07358	0.6888	0.033*

Protist **PR**

	RDA1	RDA2	r2	Pr(>r)
NO2	-0.8152	0.57917	0.0279	0.859
NO3	-0.85973	-0.51076	0.0075	0.962
NH4	-0.90989	-0.41484	0.059	0.745
Nmin	-0.94112	-0.33809	0.0103	0.944
PO4	-0.56182	0.82726	0.0777	0.634
SIO3	-0.66569	-0.74623	0.0017	0.99
TP	0.44497	-0.89554	0.2951	0.171
SA	-0.99815	0.06081	0.3438	0.122
depth	-0.7042	0.71	0.0172	0.933

Protist **BG**

	RDA1	RDA2	r2	Pr(>r)
NO2	-0.33336	-0.9428	0.5054	0.082
NO3	0.01211	-0.99993	0.5367	0.059
NH4	0.84324	-0.53754	0.3724	0.23
Nmin	0.10333	-0.99465	0.5856	0.06
PO4	-0.98304	0.18338	0.2125	0.552
SIO3	-0.66342	-0.74825	0.3848	0.224
TP	0.33286	0.94298	0.3124	0.324
SA	0.29056	0.95686	0.3297	0.292
depth	-0.0077	0.99997	0.4375	0.155

*, a significant level at 0.05. r^2, the square of the correlation index. *Pr*(>*r*), the *p-value* (number of permutations: 999). SA, salinity. TP, total phosphorus. Nmin, total inorganic nitrogen.

Table S3 The putative function of these 14 bins (strains) at KEGG level 2

Category	Bin1	Bin99	Bin114	Bin159	Bin168	Bin265	Bin275	Bin292	Bin326	Bin330	Bin338	Bin353	Bin378	Bin380
Aging	10	7	7	12	8	14	7	12	6	13	6	8	7	22
Amino acid metabolism	177	33	207	194	221	306	344	259	179	290	220	329	162	328
Biosynthesis of other secondary metabolites	14	5	30	22	28	30	28	23	25	30	12	30	20	32
Cancers: Overview	17	4	8	9	10	21	19	11	9	17	14	21	6	20
Cancers: Specific types	3	1	2	2	2	2	6	3	2	2	1	3	1	3
Carbohydrate metabolism	235	82	167	206	211	307	321	212	162	390	289	364	144	358
Cardiovascular diseases	2	0	1	0	0	1	2	1	0	0	0	0	0	0
Cell growth and death	25	12	10	17	14	23	23	17	8	13	7	22	11	18
Cell motility	31	10	46	0	6	24	11	4	6	4	15	66	2	87
Circulatory system	3	2	4	0	0	4	3	3	0	0	1	5	1	3
Digestive system	1	0	2	1	1	2	0	0	1	2	0	2	2	1
Drug resistance	22	8	39	24	19	16	12	28	13	14	4	32	11	30
Endocrine and metabolic diseases	7	4	7	3	3	9	8	6	3	8	8	11	4	8
Endocrine system	16	4	14	15	13	26	14	14	10	28	28	12	9	18
Energy metabolism	113	50	99	121	100	163	151	110	83	160	109	162	86	189
Environmental adaptation	2	0	4	3	2	1	2	2	2	1	2	6	1	4
Excretory system	0	0	0	1	1	0	1	1	1	1	2	1	1	0
Folding, sorting and degradation	39	17	36	41	35	43	34	36	28	45	23	60	31	49
Global and overview maps	1010	285	1076	1156	1078	1402	1526	1164	908	1588	993	1722	741	1723
Glycan biosynthesis and metabolism	22	6	31	35	57	26	34	28	25	25	10	44	27	34
Immune diseases	1	0	2	2	1	1	0	2	1	3	0	2	1	2
Immune system	0	0	2	2	2	0	1	2	2	2	0	2	2	2
Infectious diseases: Bacterial	14	6	19	12	10	13	13	11	6	13	11	21	7	35
Infectious diseases: Parasitic	4	0	3	0	2	4	3	1	2	3	0	2	3	3
Infectious diseases: Viral	3	0	0	0	1	0	7	3	1	0	0	1	0	0
Lipid metabolism	51	15	67	78	55	94	86	77	51	76	106	97	40	86
Membrane transport	39	23	60	62	34	51	149	71	28	158	26	191	27	186
Metabolism of cofactors and vitamins	99	21	99	117	98	112	135	102	83	112	65	164	60	174

Metabolism of other amino acids	53	7	49	34	43	75	76	45	29	46	47	69	29	74
Metabolism of terpenoids and polyketides	31	4	23	37	28	45	41	31	26	28	51	38	28	48
Nervous system	0	2	3	2	4	2	6	8	2	8	5	4	2	20
Neurodegenerative diseases	16	8	16	7	7	15	21	20	6	2	4	22	6	21
Nucleotide metabolism	83	23	74	86	93	110	106	87	82	118	48	142	82	119
Replication and repair	74	20	59	66	72	68	64	70	68	92	22	96	58	98
Signal transduction	58	17	77	59	36	65	64	61	27	62	43	99	26	184
Signaling molecules and interaction	0	0	0	0	1	0	1	0	1	0	0	1	0	0
Substance dependence	0	0	0	0	0	0	0	3	0	0	0	0	0	6
Transcription	4	2	1	4	3	8	5	3	1	6	4	8	2	4
Translation	70	66	55	75	75	94	71	51	56	100	59	90	86	77
Transport and catabolism	5	3	5	7	11	7	10	11	6	8	5	7	6	13
Xenobiotics biodegradation and metabolism	48	1	23	79	31	89	97	80	20	47	85	69	21	70

Number: the number of genes

Table S4 The sampling information

Location	Region	Lab ID	S.depth	Num.	18S ID	18S ID	18S ID	16S ID	Metagenom ID
ZS1	Pearl River estuary	ZJK-S1	0m	3	ZS1-0-1	ZS1-0-2	ZS1-0-3	ZS1-0	
ZS1	Pearl River estuary	ZJK-S1	7m	3	ZS1-20-1	ZS1-20-2	ZS1-20-3	ZS1-20	
ZS4	Pearl River estuary	ZJK-S4	0m	3	ZS4-0-1	ZS4-0-2	ZS4-0-3	ZS4-0	
ZS4	Pearl River estuary	ZJK-S4	20m	3	ZS4-20-1	ZS4-20-2	ZS4-20-3	ZS4-20	
ZF1	Pearl River estuary	ZJK-F1	0m	3	ZF1-0-2	ZF1-0-3	ZF1-20-1	ZF1-0	ZF1-0
ZF1	Pearl River estuary	ZJK-F1	16m	3	ZF1-20-1	ZF1-20-2	ZF1-20-3	ZF1-20	ZF1-20
ZF2	Pearl River estuary	ZJK-F2	0m	3	ZF2-0-1	ZF2-0-2	ZF2-0-3	ZF2-0	
ZF2	Pearl River estuary	ZJK-F2	20m	3	ZF2-20-1	ZF2-20-2	ZF2-20-3	ZF2-20	
ZF3	Pearl River estuary	ZJK-F3	0m	3	ZF3-0-1	ZF3-0-2	ZF3-0-3	ZF3-0	
ZF3	Pearl River estuary	ZJK-F3	20m	3	ZF3-20-1	ZF3-20-2	ZF3-20-3	ZF3-20	
ZF4	Pearl River estuary	ZJK-F4	0m	3	ZF4-0-1	ZF4-0-2	ZF4-0-3	ZF4-0	
ZF4	Pearl River estuary	ZJK-F4	20m	3	ZF4-20-1	ZF4-20-2	ZF4-20-3	ZF4-20	
ZF5	Pearl River estuary	ZJK-F5	0m	3	ZF5-0-1	ZF5-0-2	ZF5-0-3	ZF5-0	
ZF5	Pearl River estuary	ZJK-F5	20m	3	ZF5-20-1	ZF5-20-2	ZF5-20-3	ZF5-20	
ZF6	Pearl River estuary	ZJK-F6	0m	3	ZF6-0-1	ZF6-0-2	ZF6-0-3	ZF6-0	ZF6-0
ZF6	Pearl River estuary	ZJK-F6	20m	3	ZF6-20-1	ZF6-20-2	ZF6-20-3	ZF6-20	ZF6-20
ZF7	Pearl River estuary	ZJK-F7	0m	3	ZF7-0-1	ZF7-0-2	ZF7-0-3	ZF7-0	
ZF7	Pearl River estuary	ZJK-F7	20m	3	ZF7-20-1	ZF7-20-2	ZF7-20-3	ZF7-20	
ZF8	Pearl River estuary	ZJK-F8	0m	3	ZF8-0-1	ZF8-0-2	ZF8-0-3	ZF8-0	ZF8-0
ZF8	Pearl River estuary	ZJK-F8	20m	3	ZF8-20-1	ZF8-20-2	ZF8-20-3	ZF8-20	
ZF9	Pearl River estuary	ZJK-F9	0m	3	ZF9-0-1	ZF9-0-2	ZF9-0-3		
ZF9	Pearl River estuary	ZJK-F9	20m	3	ZF9-20-1	ZF9-20-2	ZF9-20-3	ZF9-20	ZF9-20
JN5	Beibu Gulf,	JH-F1	0m	3	JF1-0-1	JF1-0-2	JF1-0-3	JF1-0	
JN5	Beibu Gulf,	JH-F1	20m	3	JF1-20-1	JF1-20-2	JF1-20-3	JF1-20	
JN9	Beibu Gulf,	JH-F6	0m	3	JF6-0-1	JF6-0-2	JF6-0-3	JF6-0	
JN9	Beibu Gulf,	JH-F6	20m	3	JF6-20-1	JF6-20-2	JF6-20-3	JF6-20	
JS2	Beibu Gulf,	JH-F8	0m	3	JF8-0-1	JF8-0-2	JF8-0-3	JF8-0	
JS2	Beibu Gulf,	JH-F8	20m	3	JF8-20-1	JF8-20-2	JF8-20-3	JF8-20	
JS9	Beibu Gulf,	JH-S9	0m	3	JS9-0-1	JS9-0-2	JS9-0-3	JS9-0	
JS9	Beibu Gulf,	JH-S9	20m	3	JS9-20-1	JS9-20-2	JS9-20-3	JS9-20	
JN22	Beibu Gulf,	JH-N22	0m	3	JN22-0-1	JN22-0-2	JN22-0-3	JN22-0	JN22-0
JN22	Beibu Gulf,	JH-N22	20m	3	JN22-20-1	JN22-20-2	JN22-20-3	JN22-20	JN22-20
JA6	Beibu Gulf,	JH-A6	0m	3	JA6-0-1	JA6-0-2	JA6-0-3	JA6-0	JA6-0
JA6	Beibu Gulf,	JH-A6	20m	3	JA6-20-1	JA6-20-2	JA6-20-3	JA6-20	JA6-20
JA9	Beibu Gulf,	JH-A9	0m	3	JA9-0-1	JA9-0-2	JA9-0-3	JA9-0	JA9-0
JA9	Beibu Gulf,	JH-A9	20m	3	JA9-20-1	JA9-20-2	JA9-20-3	JA9-20	JA9-20
JD1	the west Guangdong waters	JH-D1	0m	3	JD1-0-1	JD1-0-2	JD1-0-3	JD1-0	JD1-0
JD1	the west Guangdong waters	JH-D1	20m	3	JD1-20-1	JD1-20-2	JD1-20-3	JD1-20	JD1-20
JD6	the west Guangdong waters	JH-D6	0m	3	JD6-0-1	JD6-0-2	JD6-0-3	JD6-0	JD6-0
JD6	the west Guangdong waters	JH-D6	20m	3	JD6-20-1	JD6-20-2	JD6-20-3	JD6-20	JD6-20

JD9	the west Guangdong waters	JH-D9	0m	3	JD9-0-1	JD9-0-2	JD9-0-3	JD9-0	JD9-0
JD9	the west Guangdong waters	JH-D9	20m	3	JD9-20-1	JD9-20-2	JD9-20-3	JD9-20	JD9-20
JH1	the east Guangdong waters	JH-H1	0m	3	JH1-0-1	JH1-0-2	JH1-0-3	JH1-0	JH1-0
JH1	the east Guangdong waters	JH-H1	20m	3	JH1-20-1	JH1-20-2	JH1-20-3	JH1-20	JH1-20
JH6	the east Guangdong waters	JH-H6	0m	3	JH6-0-1	JH6-0-2	JH6-0-3	JH6-0	JH6-0
JH6	the east Guangdong waters	JH-H6	20m	3	JH6-20-1	JH6-20-2	JH6-20-3	JH6-20	JH6-20
JH9	the east Guangdong waters	JH-H9	0m	3	JH9-0-1	JH9-0-2	JH9-0-3	JH9-0	JH9-0
JH9	the east Guangdong waters	JH-H9	20m	3	JH9-20-1	JH9-20-2	JH9-20-3	JH9-20	JH9-20

S.depth, sampling depth. Num, the number of samples.

Table S5 The barcode sequences for each sample in this study

16S			18S			18S			18S		
Sample ID	F-barcode	R-barcode	Sample ID	F-barcode	R-barcode	Sample ID	F-barcode	R-barcode	Sample ID	F-barcode	R-barcode
JA6-0	ATCGTA	TCTACC	JA6-0-1	ACAGTG	TTCAGA	ZF3-0-1	GTTTCG	CTATAC	ZF9-0-1	CTGCTG	GTGGCC
JA6-20	GGTTGT	CGATGT	JA6-0-2	GTTGAA	CATCGT	ZF3-0-2	TAACGA	ATATGT	ZF9-0-2	TAACGA	ATATGT
JA9-0	CCACAA	GCCAAT	JA6-0-3	CGTACG	GCCGCG	ZF3-0-3	AGAGTA	TTAGGC	ZF9-0-3	AGAGTA	TTAGGC
JA9-20	TCGAAG	AGTGGA	JA6-20-1	TCCCGA	ATGTCA	ZF3-20-1	GGAAGA	CTCAGA	ZF9-20-1	GGAAGA	CTCAGA
JD1-0	AGTTCC	TGAATG	JA6-20-2	CGGAAT	GGTAGC	ZF3-20-2	CTTCCA	GCCTTA	ZF9-20-2	TAACGA	ATATGT
JD1-20	GTACTT	CCAGCT	JA6-20-3	GTTTCG	CTATAC	ZF3-20-3	TGACCA	ATGCCT	ZF9-20-3	TGACCA	ATGCCT
JD6-0	CAGATC	GTGAAA	JA9-0-1	AGTTCC	TGAATG	ZF4-0-1	AGTTCC	TGAATG	JH1-0-1	CGCGGT	GAGTGG
JD6-20	TAATCG	ACTTGA	JA9-0-2	GTACTT	CCAGCT	ZF4-0-2	GTACTT	CCAGCT	JH1-0-2	TCGGCA	AGTCAA
JD9-0	ATCACG	TACAGC	JA9-0-3	CAGATC	GTGAAA	ZF4-0-3	CAGATC	GTGAAA	JH1-0-3	ATCGTA	TCTACC
JD9-20	GAGATA	CTAGCT	JA9-20-1	TAATCG	ACTTGA	ZF4-20-1	TAATCG	ACTTGA	JH1-20-1	GGTTGT	CGATGT
JF1-0	CTTCCA	GCCTTA	JA9-20-2	ATCACG	TACAGC	ZF4-20-2	ATCACG	TACAGC	JH1-20-2	CCACAA	GCCAAT
JF1-20	TGACCA	ATGCCT	JA9-20-3	GAGATA	CTAGCT	ZF4-20-3	GAGATA	CTAGCT	JH1-20-3	TCGAAG	AGTGGA
JF6-0	AGTTCC	TGAATG	JD1-0-1	CTGCTG	GTGGCC	ZF5-0-1	CGCGGT	GAGTGG	JH6-0-1	ACAGTG	TTCAGA
JF6-20	GTACTT	CCAGCT	JD1-0-2	TAACGA	ATATGT	ZF5-0-2	TCGGCA	AGTCAA	JH6-0-2	GTTGAA	CATCGT
JF8-0	CAGATC	GTGAAA	JD1-0-3	AGAGTA	TTAGGC	ZF5-0-3	ATCGTA	TCTACC	JH6-0-3	CGTACG	GCCGCG
JF8-20	TAATCG	ACTTGA	JD1-20-1	GGAAGA	CTCAGA	ZF5-20-1	GGTTGT	CGATGT	JH6-20-1	TCCCGA	ATGTCA
JH1-0	CGCGGT	GAGTGG	JD1-20-2	CTTCCA	GCCTTA	ZF5-20-2	CCACAA	GCCAAT	JH6-20-2	CGGAAT	GGTAGC
JH1-20	TCGGCA	AGTCAA	JD1-20-3	TGACCA	ATGCCT	ZF5-20-3	TCGAAG	AGTGGA	JH6-20-3	GTTTCG	CTATAC
JH6-0	ATCGTA	TCTACC	JD6-0-1	AGTTCC	TGAATG	JF6-0-1	CTGCTG	GTGGCC	JH9-0-1	CTGCTG	GTGGCC
JH6-20	GGTTGT	CGATGT	JD6-0-2	GTACTT	CCAGCT	JF6-0-2	TAACGA	ATATGT	JH9-0-2	TAACGA	ATATGT
JH9-0	CCACAA	GCCAAT	JD6-0-3	CAGATC	GTGAAA	JF6-0-3	AGAGTA	TTAGGC	JH9-0-3	AGAGTA	TTAGGC
JH9-20	TCGAAG	AGTGGA	JD6-20-1	TAATCG	ACTTGA	ZF6-0-1	ACAGTG	TTCAGA	JH9-20-1	GGAAGA	CTCAGA
JN22-0	CGCGGT	GAGTGG	JD6-20-2	ATCACG	TACAGC	ZF6-0-2	GTTGAA	CATCGT	JH9-20-2	CTTCCA	GCCTTA
JN22-20	TCGGCA	AGTCAA	JD6-20-3	GAGATA	CTAGCT	ZF6-0-3	CGTACG	GCCGCG	JH9-20-3	TGACCA	ATGCCT
JS9-0	ATCACG	TACAGC	JD9-0-1	CGCGGT	GAGTGG	JF6-20-1	GGAAGA	CTCAGA	JN22-0-1	ACAGTG	TTCAGA
JS9-20	GAGATA	CTAGCT	JD9-0-2	TCGGCA	AGTCAA	JF6-20-2	CTTCCA	GCCTTA	JN22-0-2	GTTGAA	CATCGT
ZF1-0	ATCACG	TACAGC	JD9-0-3	ATCGTA	TCTACC	JF6-20-3	TGACCA	ATGCCT	JN22-0-3	CGTACG	GCCGCG
ZF1-20	GAGATA	CTAGCT	JD9-20-1	GGTTGT	CGATGT	ZF6-20-1	TCCCGA	ATGTCA	JN22-20-1	TCCCGA	ATGTCA
ZF2-0	CGCGGT	GAGTGG	JD9-20-2	CCACAA	GCCAAT	ZF6-20-2	CGGAAT	GGTAGC	JN22-20-2	CGGAAT	GGTAGC
ZF2-20	TCGGCA	AGTCAA	JD9-20-3	TCGAAG	AGTGGA	ZF6-20-3	GTTTCG	CTATAC	JN22-20-3	GTTTCG	CTATAC
ZF3-0	ATCGTA	TCTACC	ZF1-0-1	AGTTCC	TGAATG	ZF7-0-1	AGTTCC	TGAATG	ZS1-0-1	AGTTCC	TGAATG
ZF3-20	GGTTGT	CGATGT	ZF1-0-2	GTACTT	CCAGCT	ZF7-0-2	CTGCTG	GTGGCC	ZS1-0-2	GTACTT	CCAGCT
ZF4-0	CCACAA	GCCAAT	ZF1-0-3	CAGATC	GTGAAA	ZF7-0-3	TAACGA	ATATGT	ZS1-0-3	CAGATC	GTGAAA
ZF4-20	TCGAAG	AGTGGA	JF1-0-1	CGCGGT	GAGTGG	ZF7-20-1	TAATCG	ACTTGA	ZS1-20-1	TAATCG	ACTTGA
ZF5-0	ACAGTG	TTCAGA	JF1-0-2	TCGGCA	AGTCAA	ZF7-20-2	ATCACG	TACAGC	ZS1-20-2	ATCACG	TACAGC
ZF5-20	GTTGAA	CATCGT	JF1-0-3	ATCGTA	TCTACC	ZF7-20-3	GAGATA	CTAGCT	ZS1-20-3	GAGATA	CTAGCT
ZF6-0	TCCCGA	ATGTCA	ZF1-20-1	TAATCG	ACTTGA	JF8-0-1	ACAGTG	TTCAGA	ZS4-0-1	CGCGGT	GAGTGG
ZF6-20	CGGAAT	GGTAGC	ZF1-20-2	ATCACG	TACAGC	JF8-0-2	GTTGAA	CATCGT	ZS4-0-2	TCGGCA	AGTCAA
ZF7-0	GTTTCG	CTATAC	ZF1-20-3	GAGATA	CTAGCT	JF8-0-3	CGTACG	GCCGCG	ZS4-0-3	ATCGTA	TCTACC

ZF7-20	CTGCTG	GTGGCC	JF1-20-1	GGTTGT	CGATGT	ZF8-0-1	CGCGGT	GAGTGG	ZS4-20-1	GGTTGT	CGATGT
ZF8-0	TAACGA	ATATGT	JF1-20-2	CCACAA	GCCAAT	ZF8-0-2	TCGGCA	AGTCAA	ZS4-20-2	CCACAA	GCCAAT
ZF8-20	AGAGTA	TTAGGC	JF1-20-3	TCGAAG	AGTGGA	ZF8-0-3	ATCGTA	TCTACC	ZS4-20-3	TCGAAG	AGTGGA
ZF9-20	GGAAGA	CTCAGA	ZF2-0-1	ACAGTG	TTCAGA	JF8-20-1	TCCCGA	ATGTCA	JS9-0-1	CTGCTG	GTGGCC
ZS1-0	AGTTCC	TGAATG	ZF2-0-2	GTTGAA	CATCGT	JF8-20-2	CGGAAT	GGTAGC	JS9-0-2	TAACGA	ATATGT
ZS1-20	GTACTT	CCAGCT	ZF2-0-3	CGTACG	GCCGCG	JF8-20-3	GTTTCG	CTATAC	JS9-0-3	AGAGTA	TTAGGC
ZS4-0	CAGATC	GTGAAA	ZF2-20-1	TCCCGA	ATGTCA	ZF8-20-1	GGTTGT	CGATGT	JS9-20-1	GGAAGA	CTCAGA
ZS4-20	TAATCG	ACTTGA	ZF2-20-2	CGGAAT	GGTAGC	ZF8-20-2	CCACAA	GCCAAT	JS9-20-2	CTTCCA	GCCTTA
			ZF2-20-3	GTTTCG	CTATAC	ZF8-20-3	TCGAAG	AGTGGA	JS9-20-3	TGACCA	ATGCCT

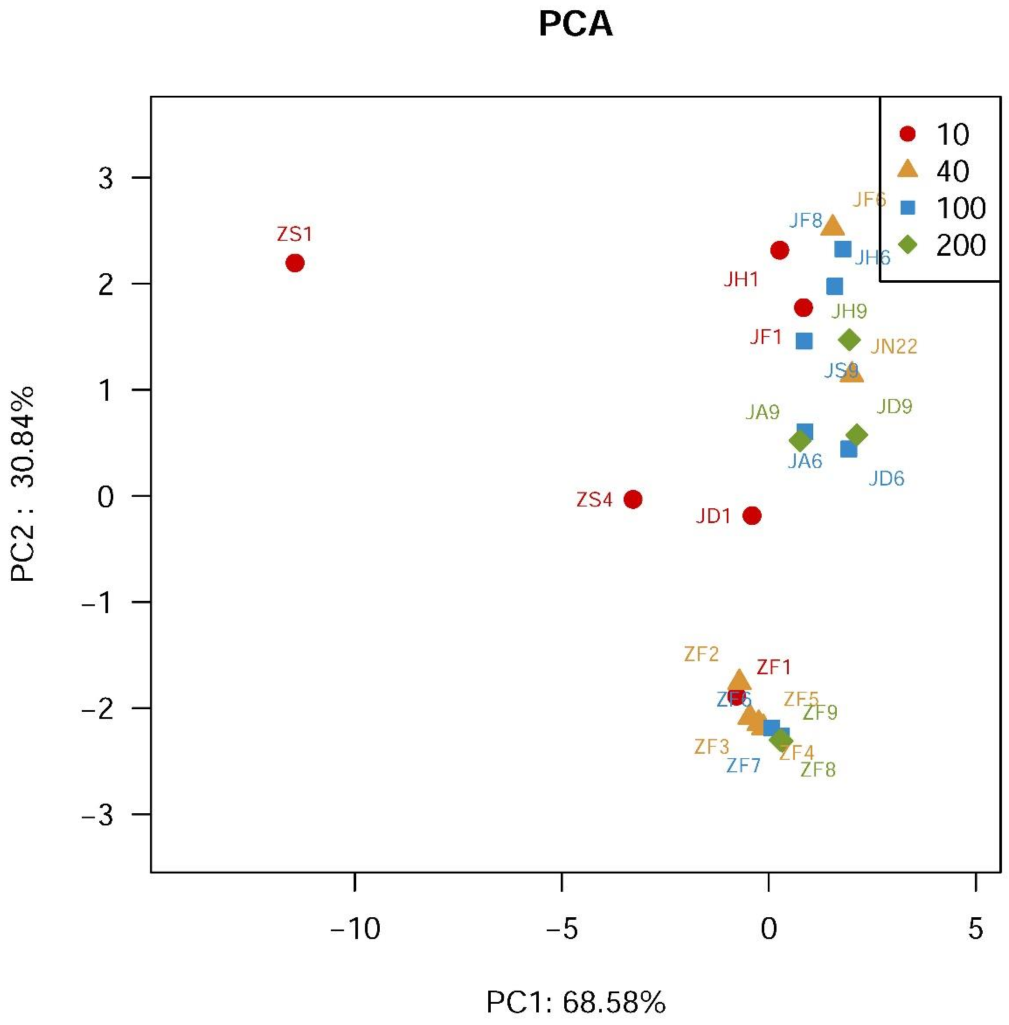

Figure S1 The PCA clustering analysis on the samples from 24 collecting site based on the Bray-Curtis distance using environmental factors measured in this study. 10, water depth at 10m. 40, water depth at 40m. 100, water depth at 100m. 200, water depth at 200m. In the left hierarchical tree, each shape represented one average sample in each sample collecting site in this study.

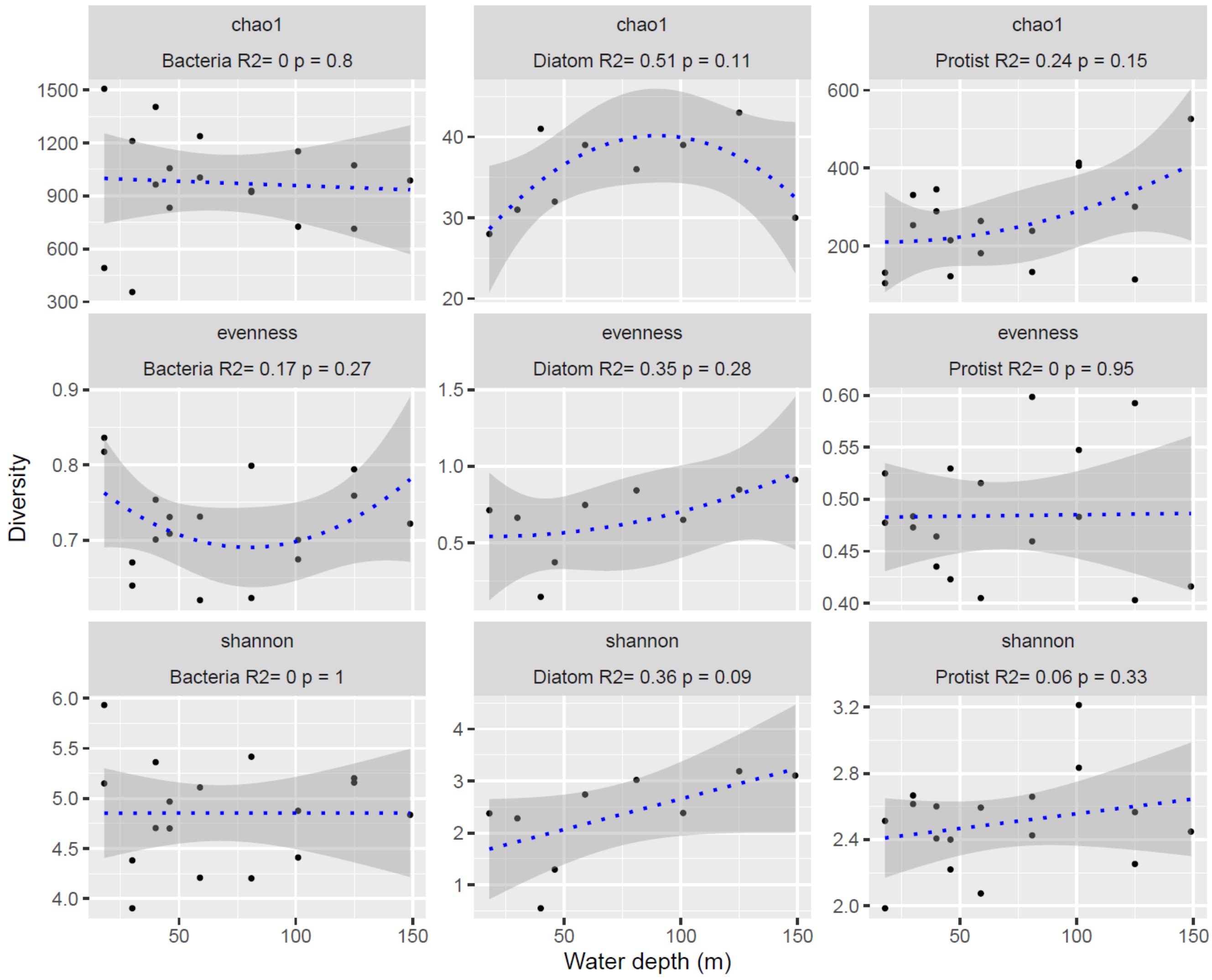

Figure S2 Water-depth patterns for bacteria, diatoms, and protists in alpha diversity in Pearl River estuary region. The relationships between water depth and alpha diversity were modeled using the linear and quadratic models. The better model was selected based on the lower value of Akaike's information criteria (Yamaoka, et al. 1978).

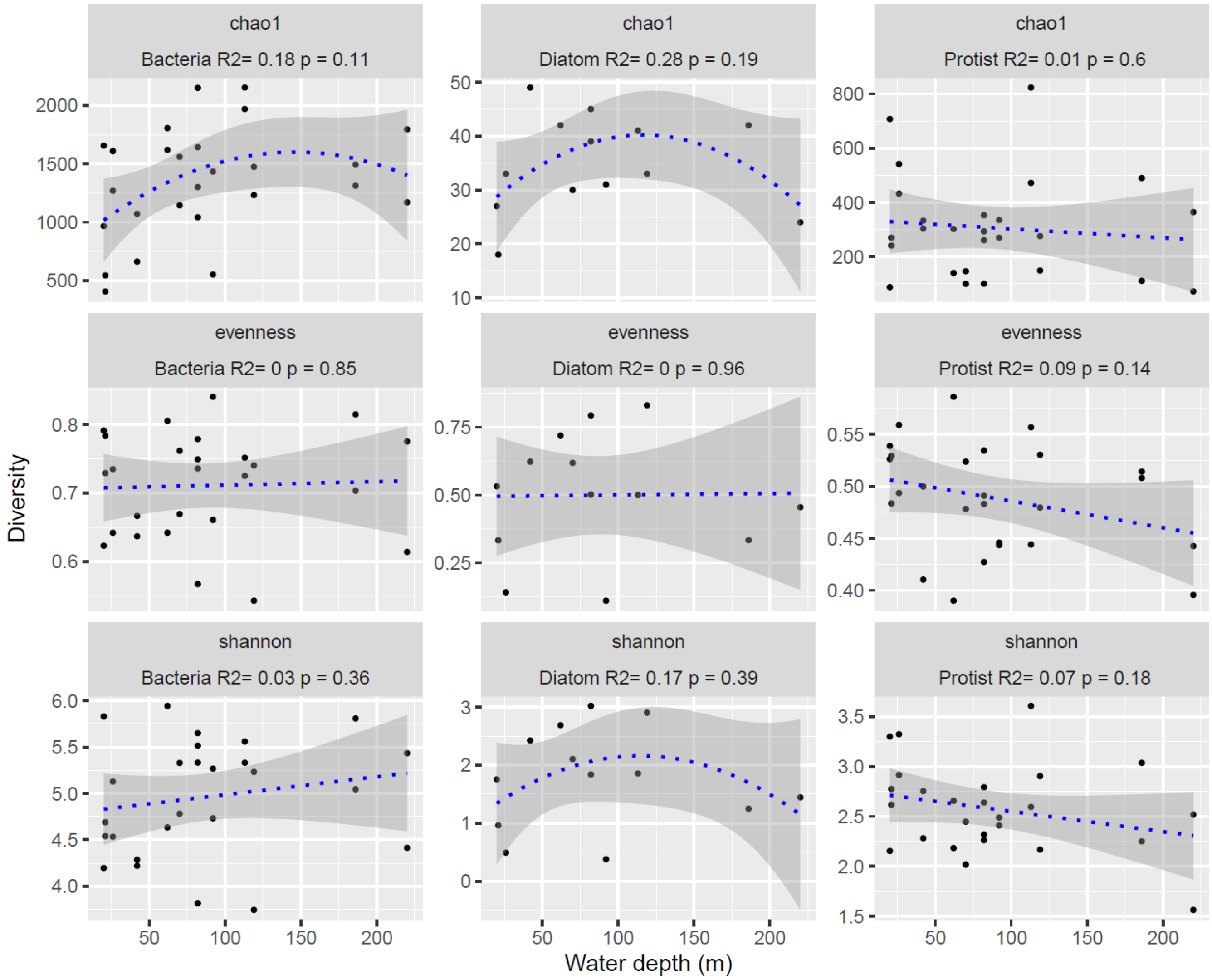

Figure S3 Water-depth patterns for bacteria, diatoms, and protists in alpha diversity in Beibu Gulf region. The relationships between water depth and alpha diversity were modeled using the linear and quadratic models. The better model was selected based on the lower value of Akaike's information criteria (Yamaoka, et al. 1978).

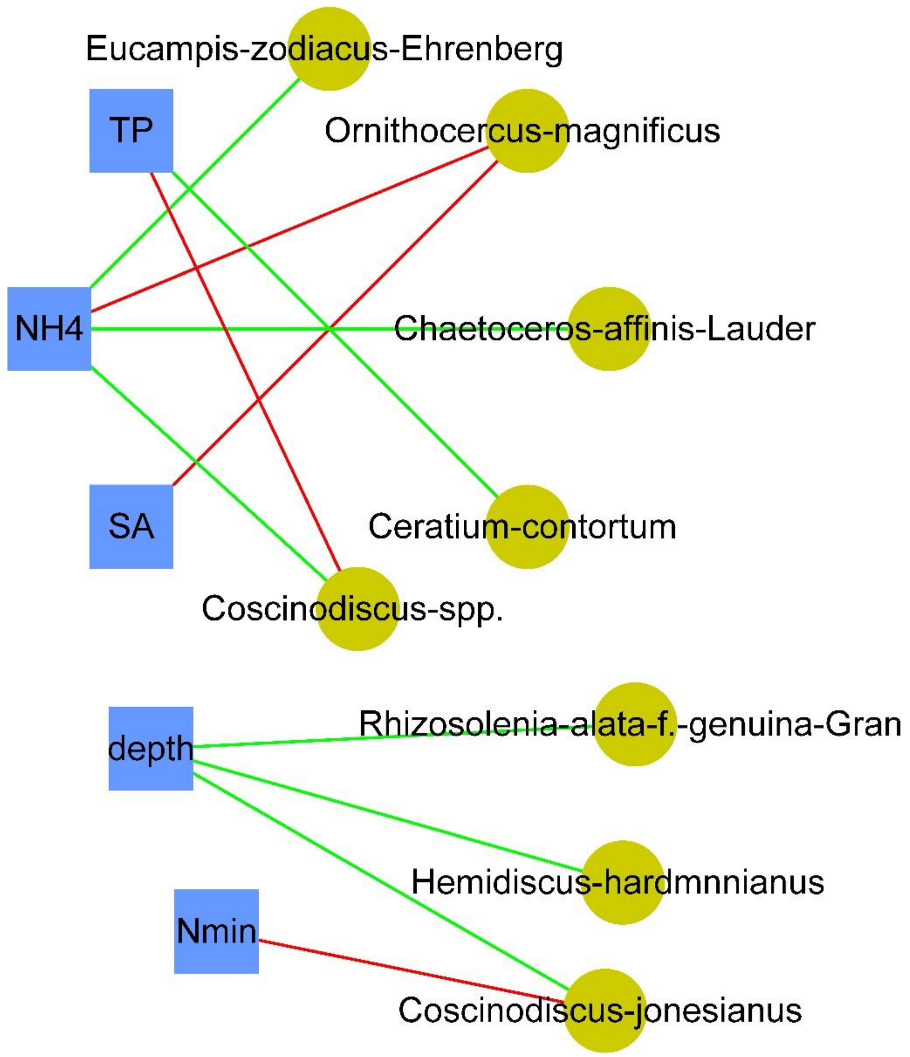

Figure S3 The significant relationship (spearman) between the diatoms and environmental factors. Green line, positively significant (0.05). Red line, negatively significant (0.05).

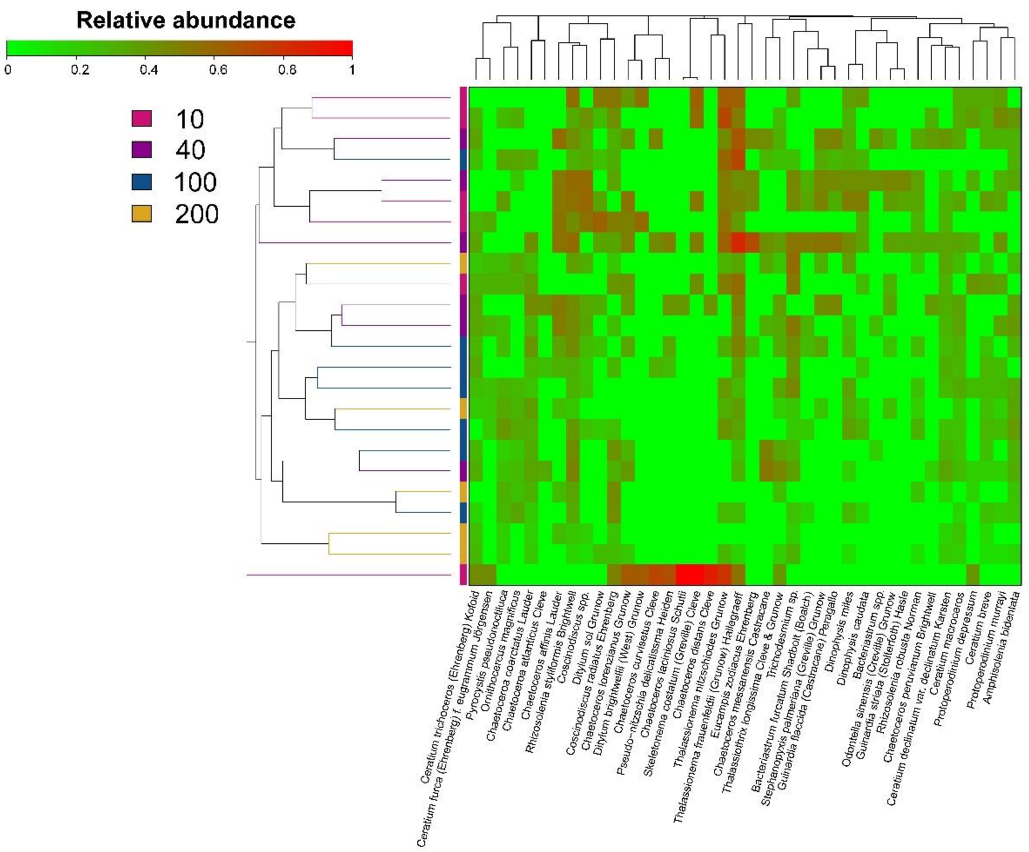

Figure S4 The heatmap of the main diatom species in this study. 10, water depth at 10m. 40, water depth at 40m. 100, water depth at 100m. 200, water depth at 200m. In the left hierarchical tree, each node represented one average sample in each sample collecting site in this study.

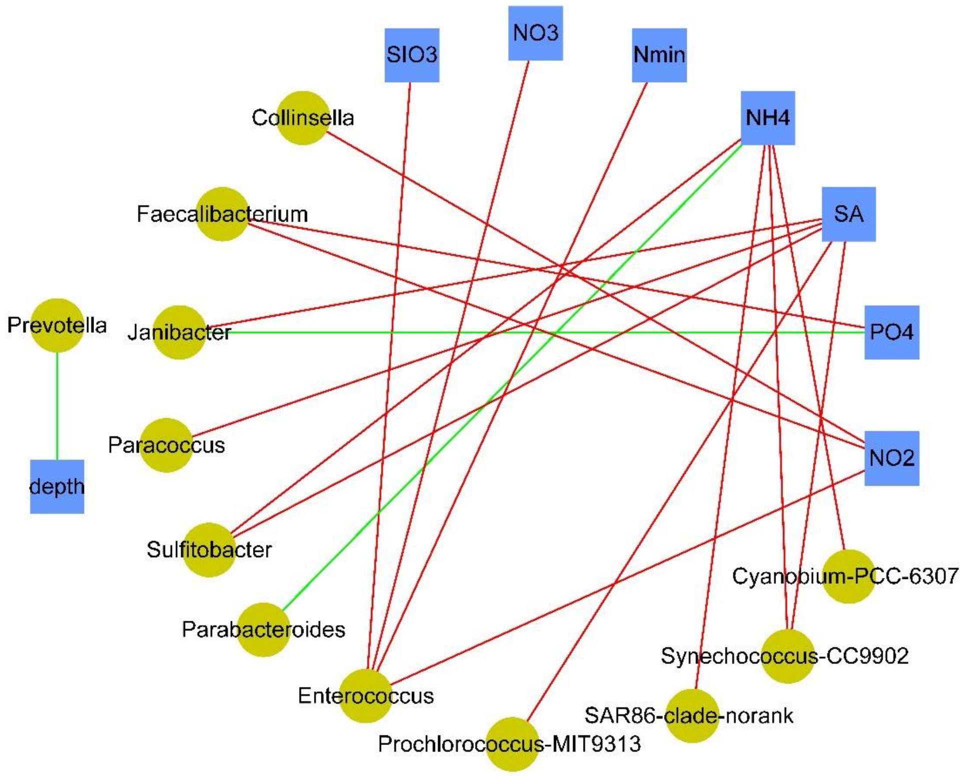

Figure S5 The significant relationship (spearman) between the bacteria and environmental factors. Green line, positively significant (0.05). Red line, negatively significant (0.05).

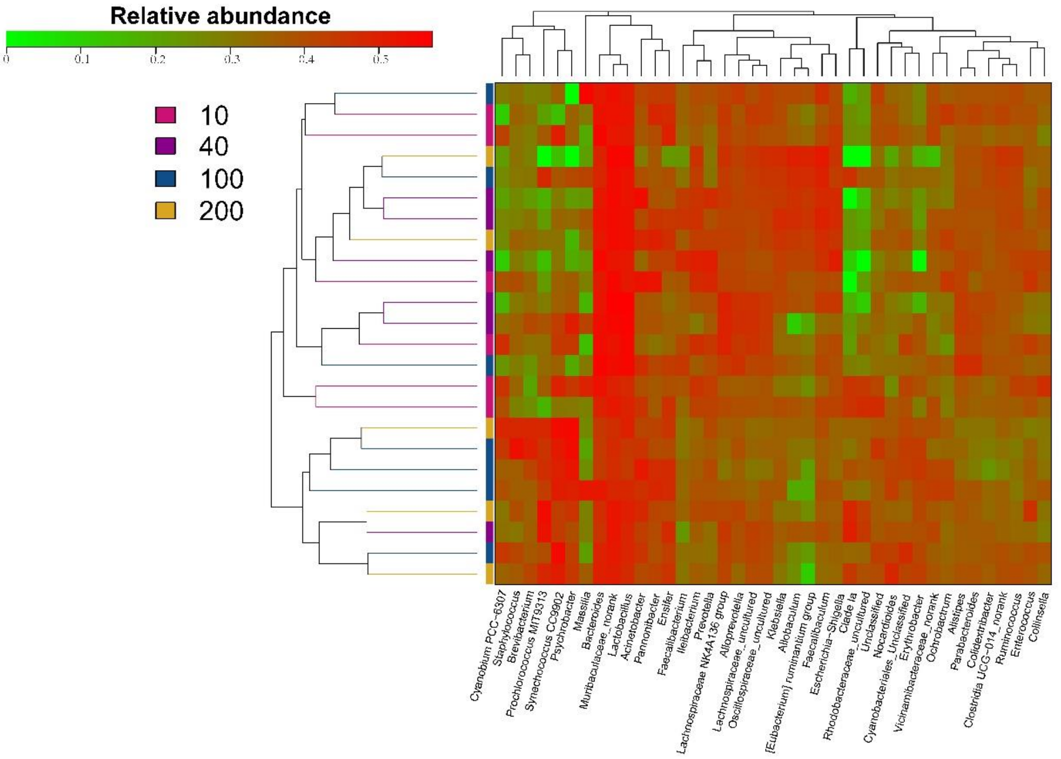

Figure S6 The heatmap of the main bacteria genera in this study. 10, water depth at 10m. 40, water depth at 40m. 100, water depth at 100m. 200, water depth at 200m. In the left hierarchical tree, each node represented one average sample in each sample collecting site in this study.

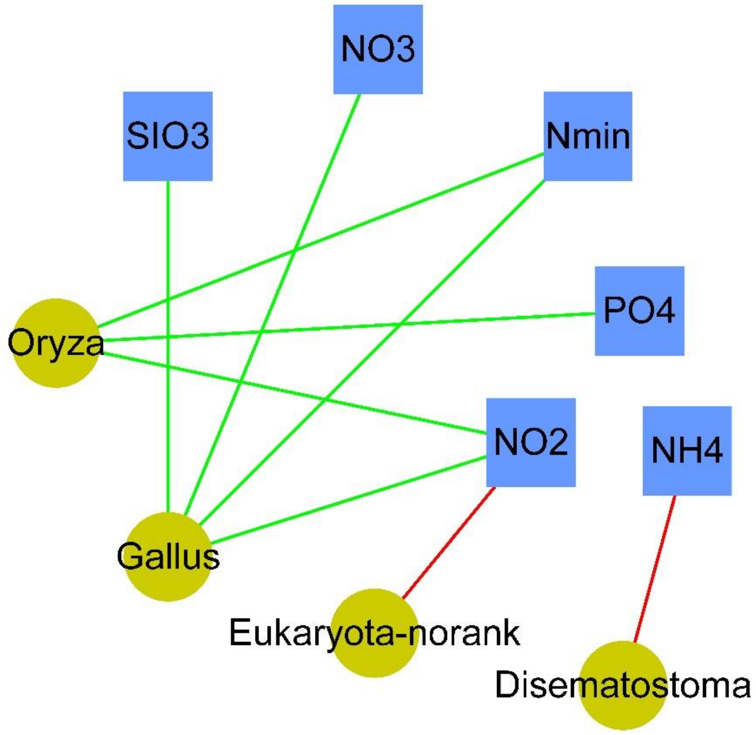

Figure S7 The significant relationship (spearman) between the protists and environmental factors. Green line, positively significant (0.05). Red line, negatively significant (0.05).

References

Yamaoka K, Nakagawa T, Uno T. 1978. Application of Akaike's information criterion (AIC) in the evaluation of linear pharmacokinetic equations. Journal of pharmacokinetics and biopharmaceutics 6:165-175.
